# Sex-dependent and muscle-specific progression of the *MYBPC1* E248K Myotrem myopathy in response to aging

**DOI:** 10.1172/jci.insight.182471

**Published:** 2025-06-26

**Authors:** Jennifer M. Mariano, Humberto C. Joca, Jacob Kallenbach, Natasha Ranu, Julien Ochala, Christopher Ward, Aikaterini Kontrogianni-Konstantopoulos

**Affiliations:** 1 Department of Biochemistry and Molecular Biology and; 2 Department of Orthopaedics, University of Maryland School of Medicine, Baltimore, Maryland, USA.; 3 Centre of Human and Applied Physiological Sciences, School of Basic and Medical Biosciences, Faculty of Life Sciences & Medicine, King’s College London, London, United Kingdom.; 4 Biomedical Institute, University of Copenhagen, Copenhagen, Denmark.

**Keywords:** Cell biology, Muscle biology, Cytoskeleton, Neuromuscular disease, Skeletal muscle

## Abstract

Dominant missense mutations in *MYBPC1*, the gene encoding the essential sarcomeric slow Myosin Binding Protein-C (sMyBP-C), are associated with Myotrem, a new, early-onset congenital myopathy characterized by muscle weakness, hypotonia, skeletal deformities, and myogenic tremor. Importantly, the clinical manifestation of Myotrem in mid- and late adulthood is unknown. Using the Myotrem *MYBPC1* E248K–knock-in (E248K-KI) murine model, we interrogated contractile performance of soleus, gastrocnemius, and tibalis anterior (TA) muscles in both male and female mice in mid- (12 months) and late (24 months) adulthood. Our findings show that the phenotypic manifestation of E248K Myotrem differs across muscle type, sex, and age. While KI soleus muscle consistently exhibited contractile impairment across both sexes and ages, KI gastrocnemius muscle displayed preserved force production. Interestingly, TA muscle showed a sex- and age-specific effect with preserved function through 12 months in both sexes and a sharp decline at 24 months solely in males. Quantitative analysis of TA sarcomeric organization uncovered structural deficits coinciding with contractile dysfunction, supporting the notion that sMyBP-C serves a primarily structural role in skeletal muscle. Collectively, our studies reveal that aging affects the E248K Myotrem myopathy in a muscle- and sex-dependent fashion and show that sarcomeric disorganization accompanies contractile deterioration in affected muscles.

## Abstract 2

Our study aims to investigate the muscle- and sex-dependent presentation and progression of Myotrem myopathy due to the inevitable process of aging and understand the underlying etiologies.

## Graphical abstract

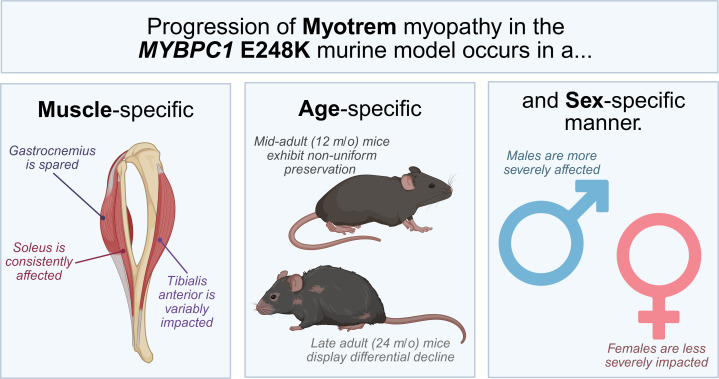

## Introduction

Myosin binding protein-C (MyBP-C) comprises a family of sarcomeric accessory proteins expressed in striated muscles with critical structural and regulatory roles. Most notably, MyBP-C contributes to thick filament assembly and maintenance, while modulating actomyosin crossbridge formation and kinetics (1–6). MyBP-C proteins are mainly composed of tandem immunoglobulin and fibronectin-III–like domains, accompanied by a proline-alanine–rich (P/A-rich) region and a highly conserved M-motif (7). Localized to the C-zone of the sarcomeric A-band, the COOH-terminus of MyBP-C is constitutively bound to the thick filament, while the NH_2_-terminus interacts dynamically with both myosin and actin filaments to regulate actomyosin crossbridge formation. There are 3 distinct isoforms in the MyBP-C family: slow skeletal, fast skeletal, and cardiac. *MYBPC1*, the gene encoding the slow skeletal isoform (sMyBP-C), is heavily spliced to give rise to at least 17 known splice variants in humans (5). These variants are coexpressed in both slow and fast skeletal myofibers to differing degrees and combinations and may have distinct structural and/or regulatory roles (3).

*MYBPC1* has garnered increasing interest due to its recognition as a myopathic gene (8). Our group has recently identified pathogenic variants in *MYBPC1* whose monoallelic presence leads to a new myopathy characterized by tremor, known as Myotrem myopathy (NIH concept ID: C5231401) or congenital myopathy-16 (CMYP16) (9, 10). The absence of neuropathy in the affected patients and the restricted expression of sMyBP-C to skeletal muscles implicate the tremor as myogenic in origin (11). In addition to tremors mainly in the upper and lower extremities — but commonly the tongue and facial muscles, as well — Myotrem carriers exhibit hypotonia, muscle weakness, and delays in gross motor milestone acquisition, including independent walking (achieved at approximately 2 years of age). Affected individuals also develop skeletal deformities presenting as spinal rigidity, scoliosis, and/or thoracic asymmetry, contractures in the elbows, and frequent respiratory infections (9).

The Myotrem phenotype manifests in infancy and progresses through childhood, entering a stabilization phase in adolescence with no further deterioration during early adulthood (9). Since the majority of diagnosed patients with Myotrem range in age between early childhood to early adulthood, it remains unclear if this stabilization phase persists in mid- and late adulthood and whether patients with Myotrem exhibit increased vulnerability to the inevitable consequences of aging.

Our lab has developed and characterized a murine Myotrem model harboring the *MYBPC1* E248K pathogenic variant (12). Homozygous inheritance of the E248K variant is neonatally lethal, likely due to respiratory failure leading to cyanosis (12). However, heterozygous animals survive and develop Myotrem myopathy that faithfully recapitulates the onset, manifestation, and progression of the disease through young adulthood, as seen in human carriers (9, 12). Accordingly, heterozygous knock-in (KI) E248K neonates exhibit a high-frequency tremor both at rest and during locomotion that subsides in young adulthood and is evident only upon intention (12). Moreover, young adult KI mice display generalized myopathy, impaired neuromuscular performance, diminished force production, and structural alterations manifesting as out-of-register sarcomeres; diffuse A-, I-, and M-bands; enlarged mitochondria; and Z-disk streaming (12). Consistent with the key structural role of sMyBP-C (1, 13), it has been postulated that these sarcomeric deficits drive the functional consequences of the E248K Myotrem myopathy (12).

Given that the E248K model reliably recapitulates Myotrem development and manifestation, we asked whether and how Myotrem myopathy progresses beyond adolescent stabilization by investigating muscle function in mid- (12 months) and late (24 months) adulthood. Intriguingly, our studies reveal that aging elicits muscle- and sex-specific alterations in the E248K Myotrem model, underscored by distinct biochemical, structural, and functional alterations. Our work therefore uncovers translational insights into the effects of biological sex and age in Myotrem presentation, severity, and underlying pathologies.

## Results

### Phenotypic and behavioral evaluation of the MYBPC1 E248K murine model in mid- and late adulthood.

To characterize the age-dependent progression of the *MYBPC1*-associated Myotrem myopathy, we utilized the established E248K murine model that we previously generated (12). No difference in survival was observed across 4 weeks to 24 months of age between WT and heterozygous KI animals for both males (Supplemental Figure 1A; supplemental material available online with this article; https://doi.org/10.1172/jci.insight.182471DS1) and females (Supplemental Figure 1B). Using a battery of phenotypic and behavioral assays, we set forth to study the manifestation and progression of the E248K Myotrem myopathy at 12 and 24 months, corresponding to mid (40–45 human years) and late (70–75 human years) adulthood, respectively (14). For each age assessed, males and females were analyzed separately, and KI animals were compared with age- and sex-matched WT littermates.

Gross neuromuscular performance assessed by an inverted hang test identified significant deficits in both male and female KI mice at 12 and 24 months compared with their sex- and age-matched WT littermates (Supplemental Figure 1, C–F), consistent with generalized neuromuscular impairment. Musculoskeletal quality and body composition were evaluated using dual energy x-ray absorptiometry (DEXA). At 12 months, KI males tended to be more kyphotic (Supplemental Figure 2, A and B) and exhibited significantly reduced body and fat mass (Supplemental Figure 2, C and D), yet higher lean mass (Supplemental Figure 2D), compared with their WT counterparts. As male E248K mice aged to 24 months, these features were largely preserved (Supplemental Figure 2, E–H). Female KI mice were likewise kyphotic at 12 months (Supplemental Figure 2, I and J) but displayed similar body (Supplemental Figure 2K), lean, and fat (Supplemental Figure 2L) mass relative to their WT littermates. At 24 months, female KI mice retained their kyphotic presentation (Supplemental Figure 2, M and N), with significant decline in body mass (Supplemental Figure 2O) underscored by lower lean and fat mass (Supplemental Figure 2P), compared with sex- and age-matched WT.

We also evaluated phenotypic behavior by observing lone mice placed in a large cage. At 12 months, WT mice of both sexes engaged in normal exploratory activities, such as roaming, rearing, sniffing, and grooming (Supplemental Videos 1 and 2). This behavior persisted at 24 months, albeit with reduced ambulation, as expected in older animals (Supplemental Videos 3 and 4). Notably, WT mice showed no signs of tremor at either 12 or 24 months, regardless of sex. KI mice of both sexes (Supplemental Videos 5 and 6) also displayed exploratory behaviors at 12 months but showed evidence of tremor during rearing. Furthermore, mid-adult KI males exhibited an affected gait, likely due to contractures in the hindlimb (Supplemental Video 5). By 24 months, KI male mice presented a pronounced tremor during rearing, grooming, and at rest (Supplemental Video 7), along with decreased ambulation and difficulty rearing. Conversely, KI female mice (Supplemental Video 8) showed a more variable phenotype at 24 months. While tremor was consistently evident, the severity along with the extent of impaired gait varied across mice. Thus, although the 2 sexes shared some deficiencies (e.g., neuromuscular impairment and tremor occurrence), they also developed distinct manifestations (e.g., skeletal abnormalities, body composition, tremor severity) as they aged.

### Evaluation of soleus contractile function in aging E248K Myotrem mice.

Muscle weakness is a major manifestation in patients with Myotrem (9, 10, 15–18). In agreement with this, our earlier work demonstrated contractile impairment in young (2- and 4-week-old) *MYBPC1* E248K mice (12). Given that patients with Myotrem reach a phase of stabilization in early adulthood, it remains unknown whether the observed functional alterations persist into mid- and late adulthood. To address this question, we first assessed contractile performance in the soleus muscle comprising part of the triceps surae (a.k.a. calf muscle) responsible for plantarflexion of the foot. Given that soleus muscle is composed of a large portion of slow-twitch fibers having abundant sMyBP-C, we considered this a sentinel muscle for tracking a pathogenic impact and its progression. Male and female WT and KI mid-adult (12-month-old) and late-adult (24-month-old) mice were analyzed separately. We utilized an ex vivo system where intact soleus muscle was placed in an oxygenated bath of Ringer’s solution and was field stimulated to induce isometric contractions, during which resulting force was measured. The force versus stimulation frequency relationship was profiled at increasing frequencies from 1 to 200 Hz, which span the range for voluntary motor activity (19–21). Contractile kinetics were evaluated at 200 Hz.

**Figure 1:**
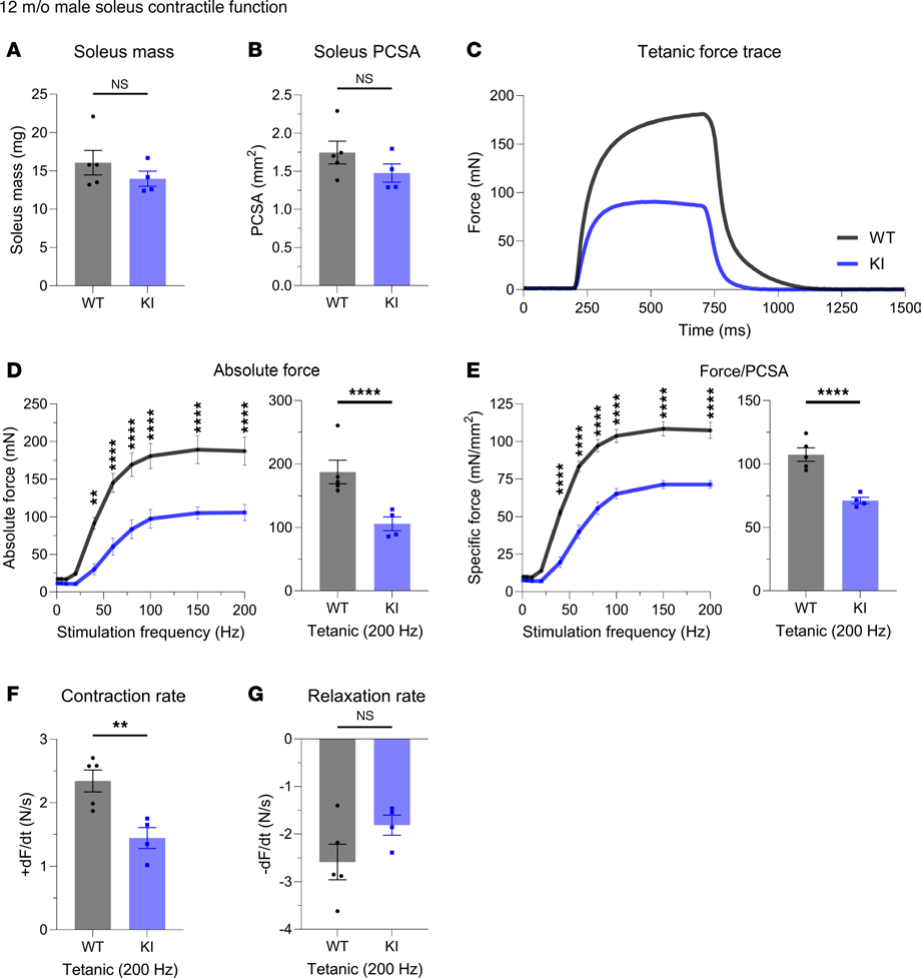
Ex vivo contractile function of 12-month-old male soleus muscle. (**A** and **B**) Soleus morphometric parameters were similar between WT and Knock-In (KI) mice in terms of mass (**A**) and physiological cross-sectional area (PCSA; **B**). (**C**) Soleus muscles from 12-month-old WT and KI male mice were mounted to a force transducer and a rigid pole on each end, and they were immersed in an ex vivo bath. Muscles were subsequently subjected to brief pulses of field stimulation between 1 and 200 Hz, and a force-frequency relationship was generated at tetanic stimulation (200 Hz). (**D**–**F**) KI male soleus muscle produced significantly reduced absolute force (**D**); specific force, defined as absolute force divided by PCSA (**E**); and rate of contraction (**F**) at 12 months compared with age- and sex-matched WT. (**G**) However, no statistical difference was observed in relaxation rate; *n* = 5 WT and *n* = 4 KI mice. Data are presented as mean ± SEM, and force traces are shown over a 1,500 msec period with a sampling rate of 125 Hz. Statistical significance was determined by 2-tailed Student’s *t* test (**A**, **B**, **F**, and **G**) and 2-way ANOVA followed by Šídák’s multiple comparisons test (**D** and **E**); NS, not significant; **P < 0.01 and ****P < 0.0001.

**Figure 2:**
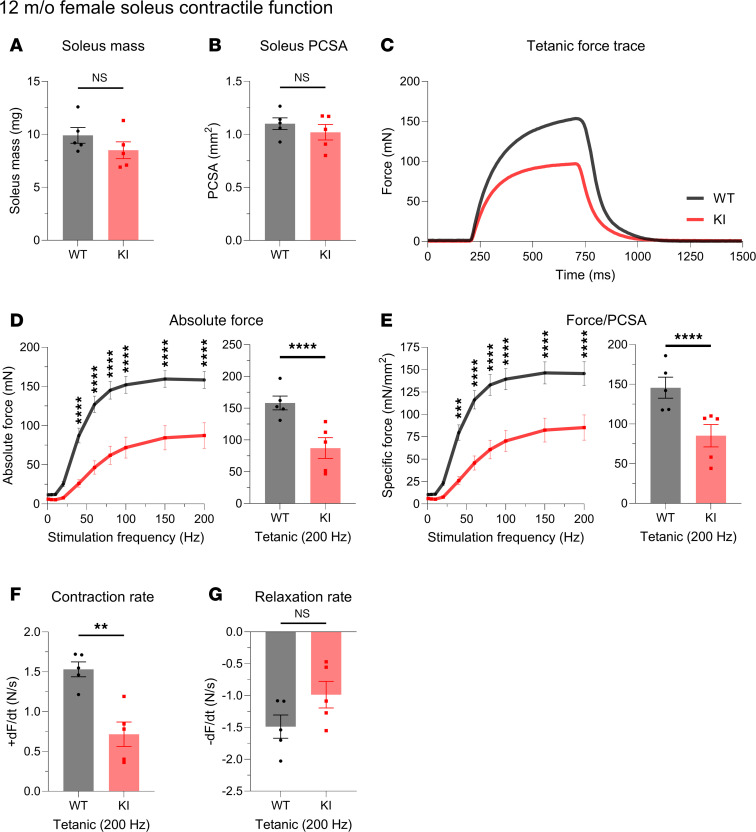
Ex vivo contractile function of 12-month-old female soleus muscle. (**A** and **B**) Morphometric evaluation of WT and KI 12-month-old females indicated similar soleus mass (**A**) and physiological cross-sectional area (PCSA; **B**). (**C**–**F**) Evaluation of ex vivo contractile function following tetanic stimulation (**C**) revealed dampened absolute force (**D**), specific force (**E**), and contraction rate (**F**) in KI female soleus muscle compared with sex- and age-matched WT. (**G**) However, changes in relaxation rate did not reach statistical significance; *n* = 5 mice per genotype. Data are presented as mean ± SEM, and force traces are shown over a 1,500 msec period with a sampling rate of 125 Hz. Statistical significance was determined by 2-tailed Student’s *t* test (**A**, **B**, **F**, and **G**) and 2-way ANOVA followed by Šídák’s multiple comparisons test (**D** and **E**); NS, not significant; **P < 0.01, ***P < 0.001, and ****P < 0.0001.

Examining WT and KI male solei at 12 months, we revealed a significant reduction in force production, despite no change in muscle mass (Figure 1, A–D). Normalizing the force to the physiological cross-sectional area (PCSA; Figure 1B), we found this force deficit to persist (Figure 1E). Quantifying the contractile kinetics, we identified a significant decline in contraction rate (Figure 1F) in the KI solei with no change in the rate of relaxation (Figure 1G). Extending this ex vivo evaluation to soleus from 12-month female WT and KI mice (Figure 2), we observed similar patterns to those of mid-adult males. Accordingly, solei mass (Figure 2A) and PCSA (Figure 2B) were comparable between WT and KI female mice at 12 months. Moreover, the KI female solei displayed similar contractile deficits with respect to tetanic absolute (Figure 2, C and D) and specific force (Figure 2E) as well as contraction kinetics (Figure 2F), while relaxation kinetics remained unaltered (Figure 2G), consistent with our findings in males.

At 24 months of age, KI male solei continued to exhibit similar mass and PCSA (Supplemental Figure 3, A and B) but impaired absolute (Supplemental Figure 3, C and D) and specific (Supplemental Figure 3E) force measurements when compared with age- and sex-matched WT, while contraction (Supplemental Figure 3F) and relaxation (Supplemental Figure 3G) kinetics appeared unchanged. The 24-month-old KI female mice also displayed statistically similar solei mass (Supplemental Figure 4A) and PCSA (Supplemental Figure 4B) to their WT counterparts, albeit with a noticeable declining trend. Accordingly, while tetanic absolute force (Supplemental Figure 4, C and D) was significantly reduced, specific force (Supplemental Figure 4E) was comparable with WT. Similar to late-adult male KI solei, contraction (Supplemental Figure 4F) and relaxation (Supplemental Figure 4G) kinetics of female KI solei were unaltered at 24 months. Thus, akin to what we previously reported in young E248K animals (12), contractile impairment of soleus muscle largely persists through mid- and late stages of adulthood for both males and females. Given that these measures were made ex vivo and independent of the nerve, the underlying mechanisms were intrinsic to the muscle fiber.

### Examination of gastrocnemius contractile function in aging E248K Myotrem mice.

We subsequently sought to evaluate contractile function of gastrocnemius, a large predominantly fast-twitch muscle located superficially to the soleus. To do so, we used an in vivo system where the mouse hindlimb was immobilized, the peroneal nerve was percutaneously stimulated between 1 and 150 Hz to evoke isometric contractions, and the resulting isometric force was quantified at increasing frequencies of stimulation.

Similar to the soleus at 12 months, the mass of gastrocnemius muscle in 12-month-old KI male mice was comparable with WT (Supplemental Figure 5A). Surprisingly, however, tetanic absolute (Supplemental Figure 5, B and C) and specific (Supplemental Figure 5D) force as well as contraction (Supplemental Figure 5E) and relaxation (Supplemental Figure 5F) kinetics were unaltered. This pattern of comparable gastrocnemius morphometry and contractile function between WT and KI mice was also apparent in 12-month-old females (Supplemental Figure 6, A–F), 24-month-old males (Supplemental Figure 7, A–F), and 24-month-old females (Supplemental Figure 8, A–E), with the exception of relaxation kinetics (Supplemental Figure 8F), which were markedly depressed in the latter group. Taken together, gastrocnemius muscle exhibited no to minimal effect compared with soleus muscle in E248K KI mice in mid- and late adulthood.

### Assessment of tibialis anterior contractile function in aging E248K Myotrem mice.

**Figure 3:**
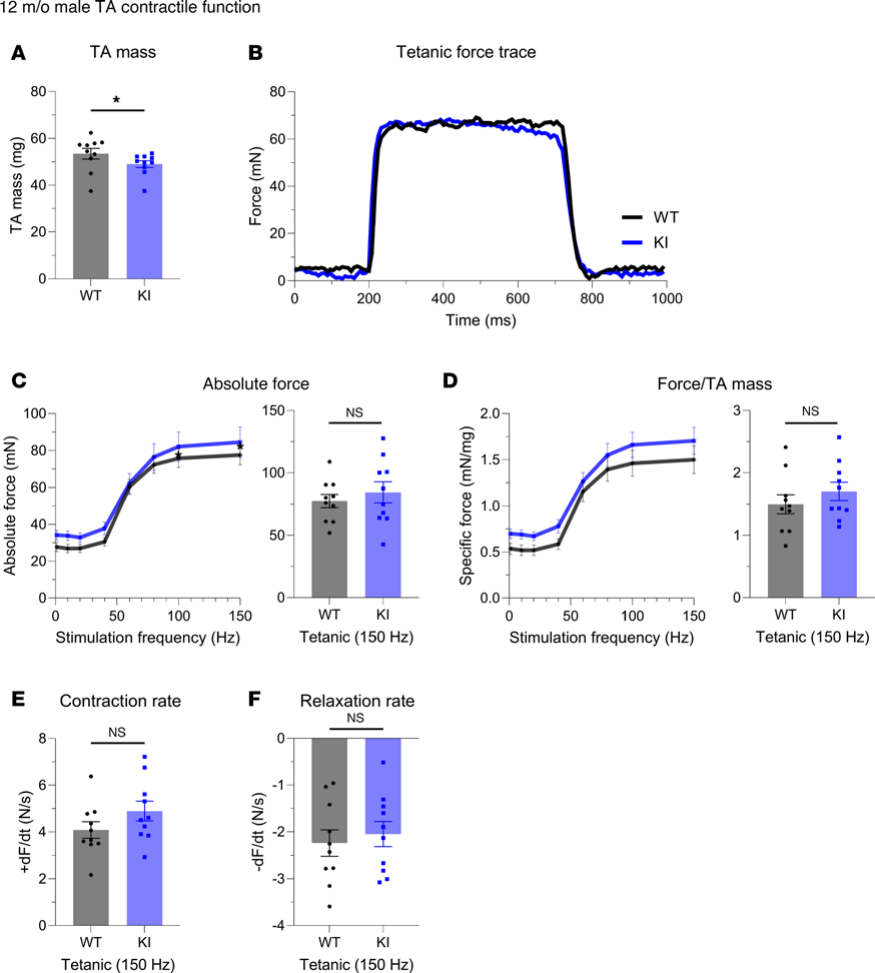
In vivo contractile function of 12-month-old male tibialis anterior muscle. Contractility was assessed using in vivo nerve-evoked isometric contractions. WT and KI mice were anesthetized, the hindlimb was immobilized, and the common peroneal nerve was percutaneously stimulated by brief (500 msec) trains of pulses delivered at 1 to 150 Hz. (**A**) Isolated tibialis anterior (TA) muscle mass was smaller in KI males when compared with WT males at 12 months. (**B**) Representative force traces of TA dorsiflexion of WT and KI 12-month-old mice resulting from 150 Hz stimulation. (**C**) Left: Force versus stimulation frequency curve. Absolute tetanic force (150 Hz; **C**, right), specific tetanic force calculated by dividing the absolute tetanic force with the muscle mass (**D**), and the rates of contraction (**E**) and relaxation (**F**) were similar between male WT and KI TA muscles at 12-months; *n* = 10 mice per genotype. Data are presented as mean ± SEM, and force traces are shown over a 1,000 msec period with a sampling rate of 125 Hz. Statistical significance was determined by 2-tailed Student’s *t* test (**E** and **F**), Mann-Whitney *U* test (**A**), and 2-way ANOVA followed by Šidák’s test for multiple comparisons (**C** and **D**); NS, not significant;*P < 0.05.

**Figure 4:**
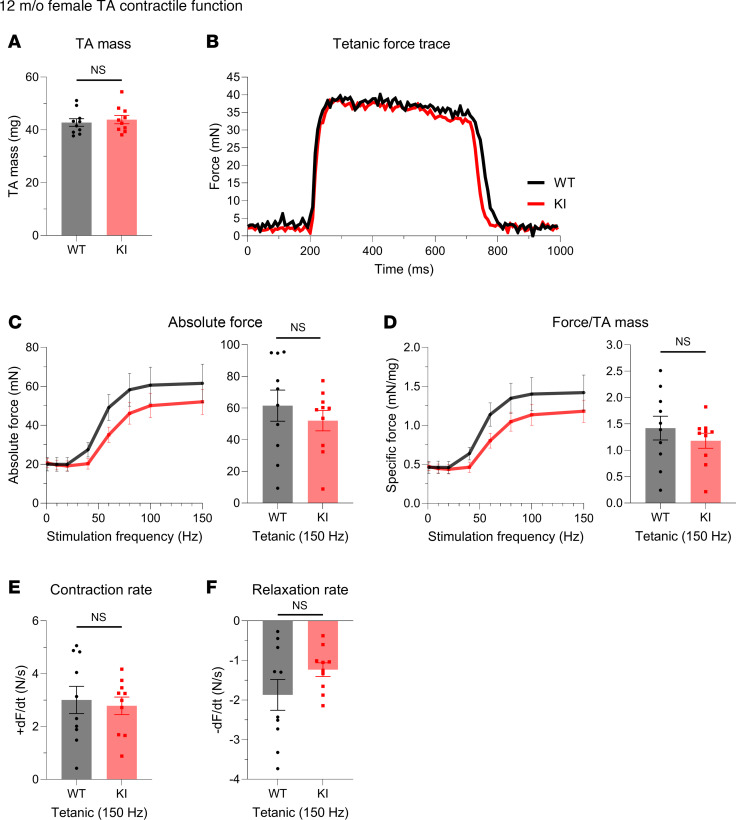
In vivo contractile function of 12-month-old female tibialis anterior muscle. Contractility was assessed using in vivo nerve-evoked isometric contractions. The common peroneal nerve was percutaneously stimulated by brief (500 msec) trains of pulses delivered at 1–150 Hz. (**A**–**F**) KI female tibialis anterior (TA) muscles exhibited comparable mass (**A**) and developed similar absolute tetanic force (**B** and **C**), specific tetanic force (**D**), and contraction (**E**) and relaxation (**F**) kinetics to WT; *n* = 10 mice per genotype. Data are presented as mean ± SEM, and force traces are shown over a 1,000 msec period with a sampling rate of 125 Hz. Statistical significance was determined by 2-tailed Student’s *t* test (**A** and **E**), 2-tailed Welch’s *t* test (**F**), and 2-way ANOVA followed by Šidák’s test for multiple comparisons (**C** and **D**). NS, not significant.

We also quantified the in vivo contractility of the tibialis anterior (TA) muscle, a large predominantly fast-twitch muscle responsible for dorsiflexion of the ankle. At 12 months, KI males exhibited significantly, yet modestly, smaller TA mass (Figure 3A) but showed comparable force production (Figure 3, B–D) and kinetics (Figure 3, E and F) to WT males. Parallel to males, 12-month-old KI females showed a similar TA morphometric change (Figure 4A) and comparable contractile function (Figure 4, B–F) to their WT counterparts.

**Figure 5:**
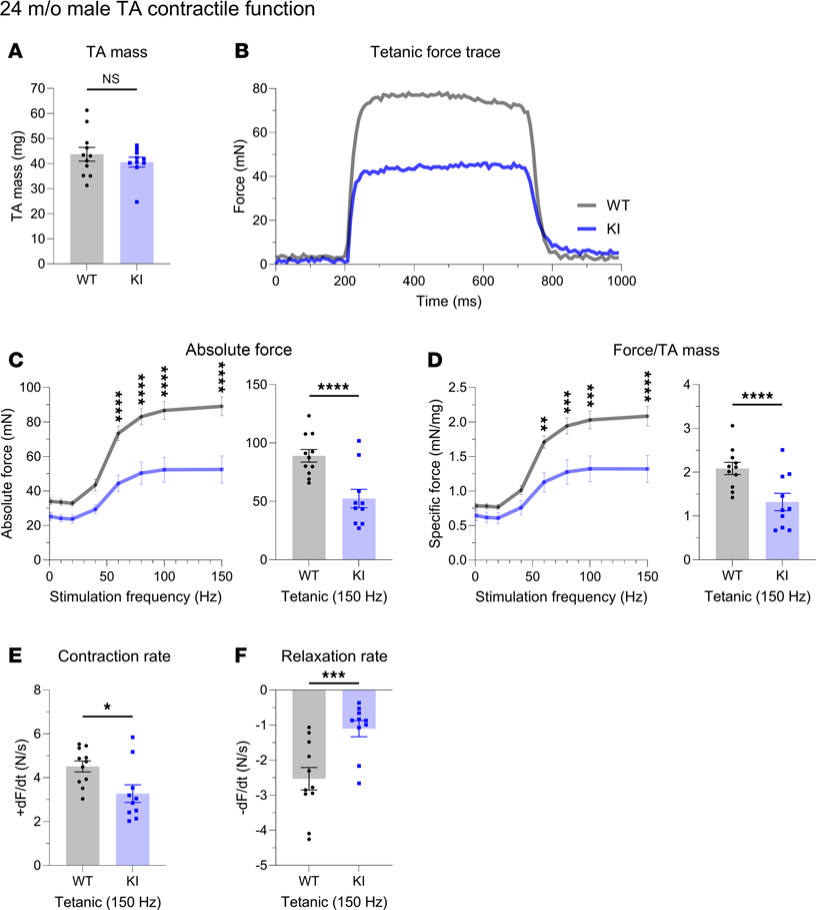
In vivo contractile function of 24-month-old male tibialis anterior muscle. Contractility was assessed using in vivo nerve-evoked isometric contractions. The common peroneal nerve was percutaneously stimulated by brief (500 msec) trains of pulses delivered at 1–150 Hz. (**A**) The mass of WT and KI tibialis anterior (TA) male muscles was similar at 24 months. (**B**–**F**) However, KI TA muscle developed significantly reduced absolute tetanic force (**B** and **C**), specific force (**D**), and contraction (**E**) and relaxation (**F**) rates compared with age- and sex-matched WT muscle; *n* = 11 WT and *n* = 10 KI mice. Data are presented as mean ± SEM, and force traces are shown over a 1,000 msec period with a sampling rate of 125 Hz. Statistical significance was determined by 2-tailed Student’s *t* test (**B**), Mann-Whitney *U* test (**A** and **C**), and 2-way ANOVA followed by Šidák’s test for multiple comparisons (**C** and **D**); NS, not significant; *P < 0.05, **P < 0.01, ***P < 0.001, and ****P < 0.0001.

**Figure 6:**
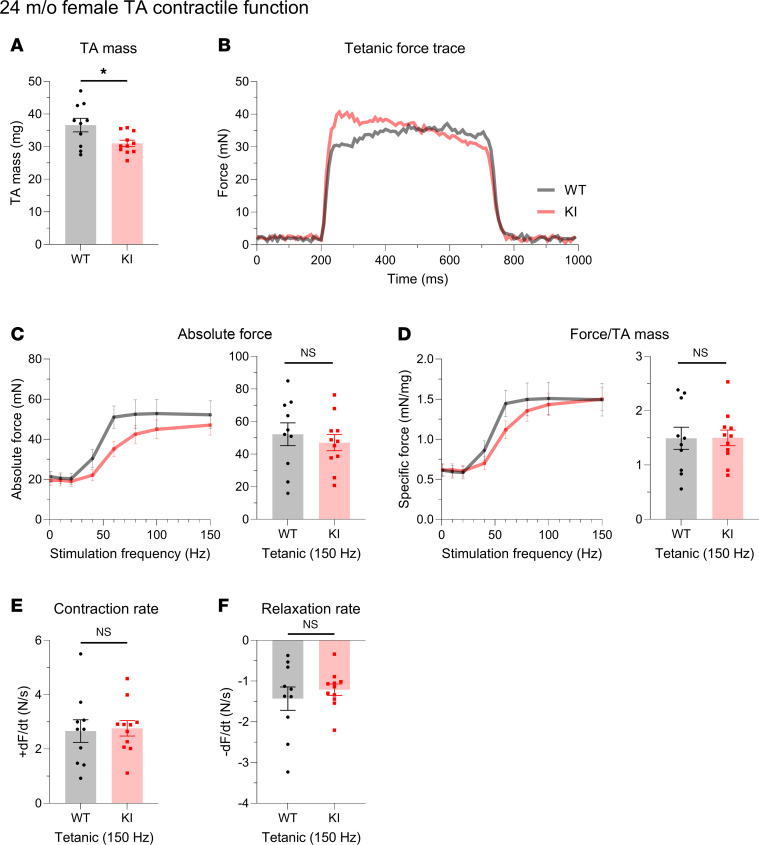
In vivo contractile function of 24-month-old female tibialis anterior muscle. Contractility was assessed using in vivo nerve-evoked isometric contractions. The common peroneal nerve was percutaneously stimulated by brief (500 msec) trains of pulses delivered at 1 to 150 Hz. (**A**) At 24 months, KI female tibialis anterior (TA) muscles exhibited smaller muscle mass compared with WT. (**B**–**F**) Absolute tetanic force (**B** and **C**), specific force (**D**), and the rates of contraction (**E**) and relaxation (**F**) were comparable between KI and WT muscles; *n* = 10 WT and *n* = 11 KI mice. Data are presented as mean ± SEM, and force traces are shown over a 1,000 msec period with a sampling rate of 125 Hz. Statistical significance was determined by 2-tailed Student’s *t* test (**E**), 2-tailed Welch’s *t* test (**A** and **F**), and 2-way ANOVA followed by Šidák’s test for multiple comparisons (**C** and **D**); NS, not significant; *P < 0.05.

KI male mice at 24 months of age deviated from the general pattern of contractile preservation observed in mid-adulthood. While similarities in TA mass were conserved (Figure 5A), contractile performance following tetanic stimulation (Figure 5B) was drastically impaired in 24-month-old KI TA muscle compared with WT, as evidenced by significant depression in both absolute (Figure 5C) and specific (Figure 5D) force as well as contractility kinetics (Figure 5, E and F). Interestingly, this decline in male KI TA function in late adulthood did not carry over to female KI TA muscle. Despite the smaller mass of female KI TA muscle compared with WT at 24 months (Figure 6A), contractile stabilization was evident (Figure 6, B–F). Thus, while both KI males and females exhibited similar TA contractile function to their WT counterparts in mid-adulthood, by late adulthood males, but not females, showed a sharp decline in both force production and contractility kinetics.

Collectively, these findings indicate that Myotrem soleus muscle consistently displayed contractile deficits, gastrocnemius muscle reliably exhibited functional preservation, whereas TA muscle showed a differential impairment profile influenced by both sex and age.

### Evaluation of sarcomeric structure, sMyBP-C expression, and myosin relaxation state of male and female E248K TA muscles.

To investigate potential cellular alterations underlying the unique sex- and age-dependent phenotype of KI TA muscle, we employed a variety of biochemical and imaging assays. Immunoblotting coupled with densitometry was used to evaluate the relative expression levels of sMyBP-C in TA muscle. To gain insights into biochemical alterations potentially preceding the observed functional deficits, we included 6-month-old mice in our analysis. sMyBP-C expression was nondifferent between male WT and KI TA muscles at 6 (Supplemental Figure 9A), 12 (Supplemental Figure 9B), or 24 (Supplemental Figure 9C) months. In contrast, female KI TA muscles exhibited an upregulation in sMyBP-C levels at 6 months (Supplemental Figure 9D), shifting to a notable decrease by 12 months (Supplemental Figure 9E); however, this reduction did not achieve statistical significance at 24 months, likely due to the large variability in sMyBP-C expression among KI female muscles (Supplemental Figure 9F).

Since MyBP-C is implicated in the regulation of the myosin relaxation state (22–24), we utilized Mant-ATP chase single nucleotide turnover assays to determine the ratio of myosin heads in disordered-relaxed (DRX; defined by faster ATPase activity and higher force-generating potential) versus super-relaxed (SRX; defined by slower ATPase activity and lower force-generating potential) states. Similar ratios (P) and lifetimes (T) of myosin heads in DRX (P_1_, T_1_) versus SRX (P_2_, T_2_) states were measured between WT and KI TA myofibers from 12-month-old male mice (Supplemental Figure 10, A–E), both of which persisted at 24 months (Supplemental Figure 10, F–J). Conversely, myofibers from 12-month-old KI females exhibited a significant increase of myosin heads in the energy-consuming DRX state (Supplemental Figure 10, K and L) and a reciprocal decrease in the energy-saving SRX state (Supplemental Figure 10, K and M) compared with sex- and age-matched WT. T_1_ and T_2_ lifetimes in 12-month-old KI females, however, remained unaltered (Supplemental Figure 10, N and O). These results are consistent with the downregulation of sMyBP-C expression in mid-adult KI females (Supplemental Figure 9E), since MyBP-C is thought to stabilize the SRX confirmation of myosin (25). By 24 months, this shift in myosin populations is less pronounced (Supplemental Figure 10P), leading to no significant differences in P_1_ (Supplemental Figure 10Q) and P_2_ (Supplemental Figure 10R) ratios between WT and KI female myofibers, and comparable T_1_ and T_2_ lifetimes (Supplemental Figure 10, S and T). Therefore, in KI females, but not males, alterations in sMyBP-C expression and, consequently, myosin relaxation state could stabilize TA contractility in mid-adulthood.

### Quantitative analysis of sarcomeric structure of mid- and late adult male and female TA.

**Figure 7:**
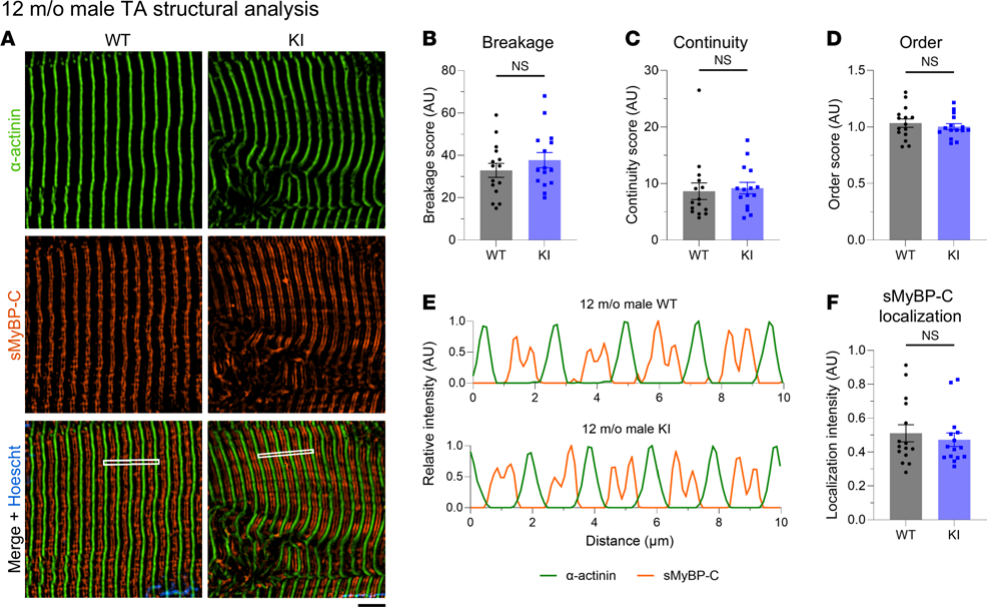
Quantitative evaluation of sarcomeric structure in 12-month-old male tibialis anterior muscle. (**A**) Representative images of tibialis anterior (TA) muscle sections labeled for α-actinin and slow Myosin Binding Protein-C (sMyBP-C). Scale bar: 5 μm. (**B**–**D**) Quantification of overall myofibrillar structure performed on α-actinin–stained images revealed comparable breakage (**B**), continuity (**C**), and order (**D**) scores between WT and KI TA male muscles at 12 months. (**E**) Linear profiles of α-actinin and sMyBP-C relative intensities confirmed their alternating distribution, corresponding to Z-disk and C-zone localization, respectively; the regions of interest (ROIs) used for analysis are denoted with a white rectangle in the merged images. (**F**) sMyBP-C exhibited similar localization scores in WT and KI TA male muscles at 12 months, as determined via Fast Fourier Transform (FFT) analysis. *n* = 3 mice per genotype and *n*′ = 5 images per muscle. Data are presented as mean ± SEM, and statistical significance was determined with 2-tailed Student’s *t* test (**B** and **D**) or Mann-Whitney *U* test (**C** and **F**). NS, not significant.

**Figure 8:**
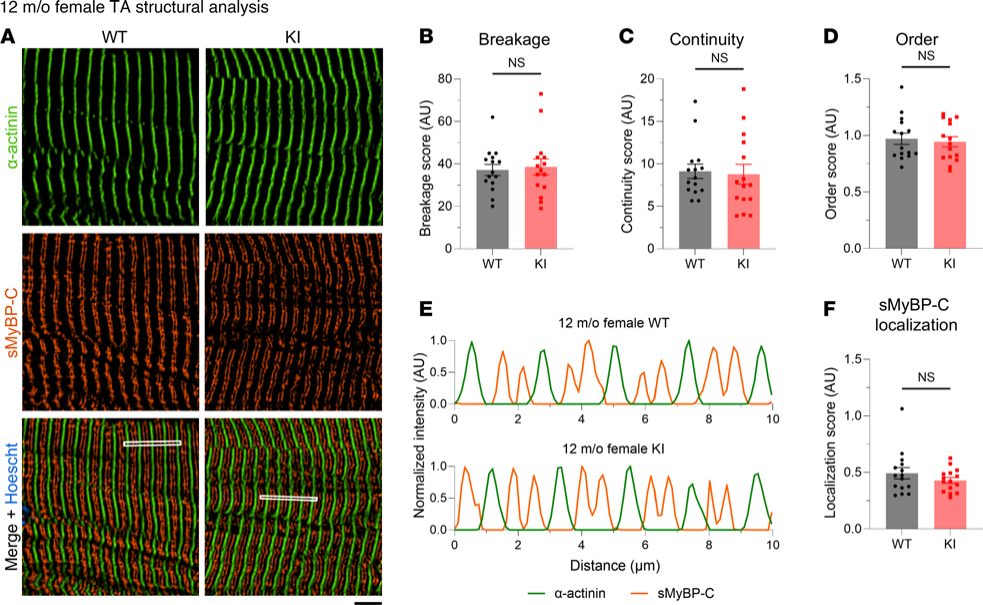
Quantitative evaluation of sarcomeric structure in 12-month-old female tibialis anterior muscle. (**A**) Representative images of tibialis anterior (TA) muscle sections immunostained for α-actinin and slow Myosin Binding Protein-C (sMyBP-C). Scale bar: 5 μm. (**B**–**D**) At 12 months, KI female TA muscle exhibits similar levels of breakage (**B**), continuity (**C**), and order (**D**) to age- and sex-matched WT tissue. (**E**) Relative intensity profiles of α-actinin and sMyBP-C confirmed their alternating distribution, corresponding to Z-disk and C-zone distribution, respectively; the regions of interest (ROIs) used for analysis are indicated with a white rectangle in the merged images. (**F**) sMyBP-C exhibited comparable localization scores between WT and KI TA female muscles at 12 months, as calculated via Fast Fourier Transform (FFT) analysis. *n* = 3 mice per genotype and *n’* = 5 images per muscle. Data are presented as mean ± SEM, and statistical significance was determined by 2-tailed Student’s *t* test (**B** and **D**) and Mann-Whitney *U* test (**C** and **F**) NS, not significant.

Given the essential role of sMyBP-C in myofibrillar assembly and maintenance (1, 13), we quantitatively assessed the structural organization of TA tissue of mid- and late-adult male and female mice. TA muscle sections were immunostained for the Z-disk marker α-actinin and sMyBP-C (Supplemental Figure 11, A–F) and were scored for levels of sarcomeric breakage, continuity, and order along with sMyBP-C localization (Supplemental Figure 11, G–N). The breakage, continuity, and order scores were comprehensively evaluated to determine whether tissues reflected prototypic sarcomeric organization (Supplemental Figure 12A) or distinct patterns of sarcomeric abnormalities, including lateral misalignment, sarcomeric continuum, disordered organization, and chaotic organization (Supplemental Figure 12 B–E). Although areas of disorganization were observed in KI TA male muscles at 12 months of age (Figure 7A), quantification of overall sarcomeric structure revealed comparable levels of myofibrillar breakage (Figure 7B), continuity (Figure 7C), and order (Figure 7D) between WT and KI, indicating a preservation of prototypic sarcomeric organization (Supplemental Figure 12A). Relatedly, sMyBP-C was properly localized to the C-zone of A-bands in mid-adult WT and KI male TA muscles (Figure 7, A, E, and F). Similarly, 12-month female TA muscles (Figure 8A) also displayed similar levels of myofibrillar breakage (Figure 8B), continuity (Figure 8C), and order (Figure 8D), while sMyBP-C was properly targeted to the C-zone (Figure 8, A, E, and F). Therefore, sarcomeric structure appeared preserved in KI male and female TA muscles in mid-adulthood, consistent with the contractile stabilization observed at this time point.

**Figure 9:**
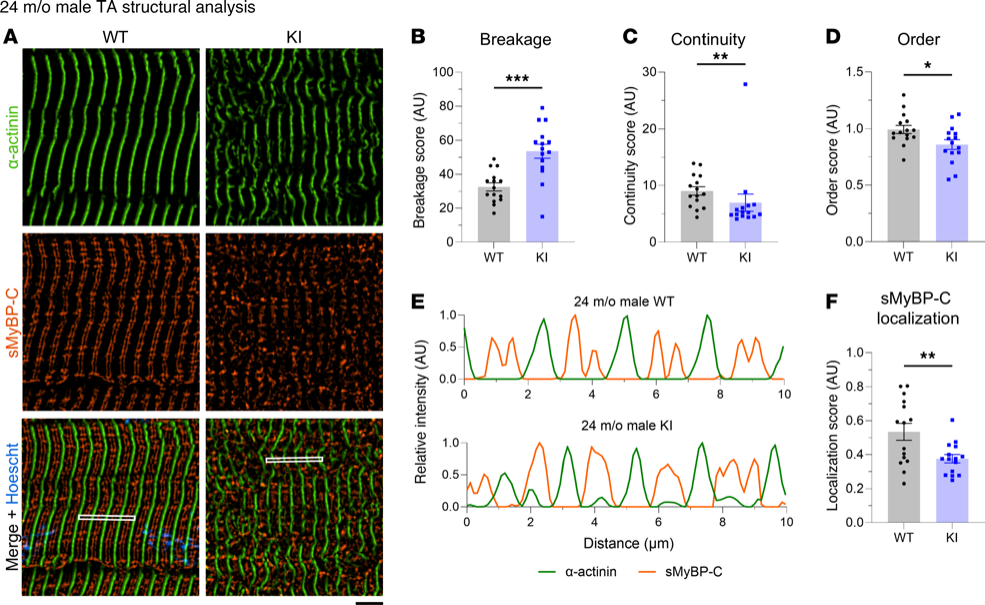
Quantitative evaluation of sarcomeric structure in 24-month-old male tibialis anterior muscle. (**A**) Representative images of tibialis anterior (TA) muscle sections colabeled for α-actinin and slow Myosin Binding Protein-C (sMyBP-C). Scale bar: 5 μm. (**B**–**D**) Contrary to 12 months, at 24 months KI male TA muscles displayed significantly increased breakage (**B**) and markedly decreased continuity (**C**) and order (**D**) scores compared with sex- and age-matched WT tissue. (**E** and **F**) Consistently, sMyBP-C failed to occupy the typical C-zone doublets, as shown in linear plots of the relative intensity profiles of α-actinin and sMyBP-C (**E**; the ROI used for analysis are outlined by a white rectangle in the merged images), and the significantly reduced sMyBP-C localization score of KI TA muscles compared with WT (**F**); *n* = 3 mice per genotype and *n’* = 5 images per muscle. Data are presented as mean ± SEM, and statistical significance was determined by 2-tailed Student’s *t* test (**B** and **F**) and Mann-Whitney *U* test (**C**); *P < 0.05, **P < 0.01, and ***P < 0.001.

**Figure 10:**
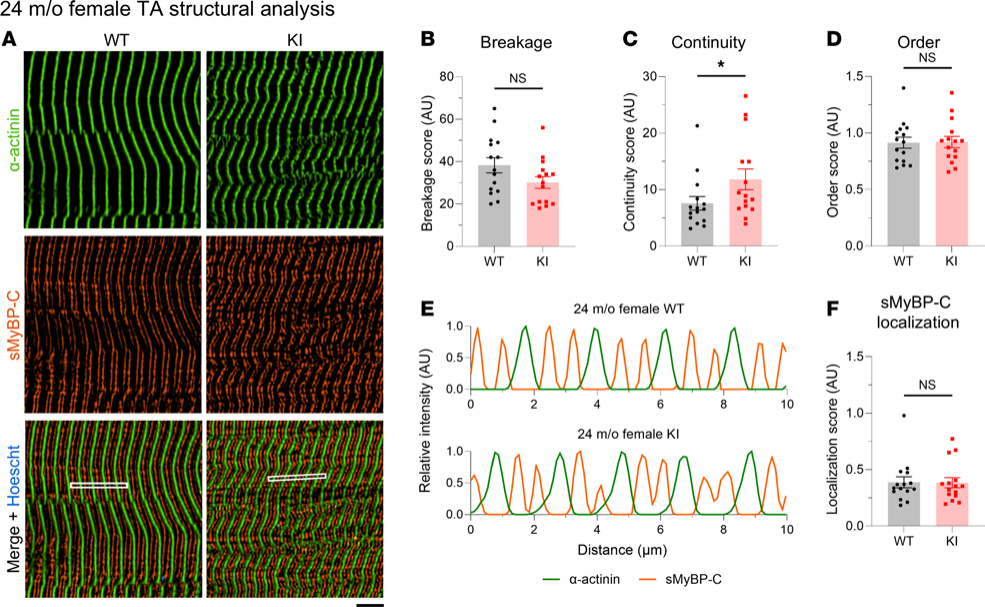
Quantitative evaluation of sarcomeric structure in 24-month-old female tibialis anterior muscle. (**A**) Representative images of tibialis anterior (TA) tissue sections were immunolabeled for α-actinin and slow Myosin Binding Protein-C (sMyBP-C). Scale bar: 5 μm. (**B**–**D**) Relative to WT female TA muscles, KI muscles exhibited comparable breakage (**B**), increased continuity (**C**), and similar order (**D**) scores. (**E** and **F**) Moreover, sMyBP-C assumed its typical doublet distribution at sarcomeric C-zones in both WT and KI TA muscles, as shown in linear plots of the relative intensity profiles of α-actinin and sMyBP-C (**E**; the ROI used for analysis are marked with a white rectangle in the merged images), and similar sMyBP-C localization scores (**F**); *n* = 3 mice per genotype and *n’* = 5 images per mouse. Data are presented as mean ± SEM, and statistical significance was determined by 2-tailed Student’s *t* test (**B** and **D**) and Mann-Whitney *U* test (**C** and **F**); NS, not significant;*P < 0.05.

Interestingly, examination of the structural organization of 24-month-old KI male TA muscles, which exhibited contractile impairment (Figure 5), uncovered severe structural deficits (Figure 9A). Late-adult male TA muscle exhibited higher breakage (Figure 9B), along with lower continuity (Figure 9C) and order (Figure 9D) scores compared with sex- and age-matched WT tissue, indicative of a pattern of chaotic organization (Supplemental Figure 12E). Consistently, sMyBP-C assumed a disordered distribution in KI TA male muscle at 24 months (Figure 9A), failing to assemble in the typical C-zone doublets seen in WT muscle (Figure 9, A and E). This was further substantiated by the markedly lower localization score of sMyBP-C in 24-month-old male KI TA muscle compared with sex- and aged-matched WT (Figure 9F). Notably, TA muscles from 24-month-old females, which conversely showed persistent contractile stabilization through late adulthood, retained sarcomeric structural integrity (Figure 10A), as quantified by breakage (Figure 10B) and order (Figure 10D) scores, which were comparable with sex- and age-matched WT. Interestingly though, late-adult female TA muscles displayed a higher continuity score relative to WT (Figure 10C), indicative of a sarcomeric continuum, characterized by a wavelike pattern of structural (mal)adaptation (Supplemental Figure 12C). Moreover, sMyBP-C properly occupied the sarcomeric C-zone in 24-month female TA muscles of both genotypes (Figure 10, A and E), as reflected by their similar localization scores (Figure 10F). Taken together, our observations indicate that the degree of structural (dis)organization in E248K TA muscles aligns with contractile (dys)function, in an age- and sex-dependent fashion, underscoring the critical role of sMyBP-C in upholding sarcomeric structure/function.

## Discussion

*MYBPC1* is an essential sarcomeric gene encoding sMyBP-C that has both structural and regulatory roles (26). Pathogenic *MYBPC1* variants have been previously associated with the development of severe and lethal forms of arthrogryposis multiplex congenita (8, 27–29) and, more recently, with a new myopathy referred to as Myotrem. Myotrem is characterized by early-onset generalized muscle weakness, hypotonia, skeletal deformities manifesting as spinal rigidity and scoliosis, dysmorphia, respiratory deficiencies, and most notably a postural tremor of myogenic origin (9, 10, 15, 18). Identification of Myotrem in the human population occurred in a multigenerational Latvian family heterozygous for the E248K variant, residing in the NH_2_-terminal M-motif of the protein that is implicated in the dynamic binding to both myosin and actin filaments (9). The index patient, a 30-year-old man at the time of diagnosis, exhibited the characteristic Myotrem phenotype that peaked in adolescence, reaching a phase of stabilization in early adulthood with no evidence of further deterioration (9). To study the E248K Myotrem myopathy in vivo, our group developed the relevant KI murine model. Importantly, the E248K model faithfully recapitulates Myotrem presentation postnatally and in early adulthood (12), establishing it as a reputable proxy to investigate the disease pathogenesis and progression.

Herein we took advantage of the E248K murine model to examine how Myotrem myopathy progresses in different muscle types across sexes in response to aging. Our findings reveal a muscle-specific presentation of Myotrem whereby contractile performance of distinct distal leg muscles is differentially affected. KI soleus muscle is most prominently affected, displaying deficits in force production and contractility kinetics at both 12 and 24 months, regardless of sex. In contrast, KI gastrocnemius muscle shows comparable contractile function to age- and sex-matched WT in mid- and late adulthood. TA muscle, however, exhibits a unique manifestation; while KI TA muscle displays contractile stabilization in both sexes at 12 months, it exhibits a sharp decline in force production in males, but not females, at 24 months, coincident with structural impairment. The consistent alignment of TA (dys)function and structural (dis)organization across sexes and time points reinforces the prevailing notion of sMyBP-C’s “structure before function” role in skeletal muscle (1, 13), distinguishing it from cMyBP-C (30, 31) and fMyBP-C (32) recognized for their principal regulatory roles.

Interestingly, closer inspection of 12-month-old E248K male TA muscle reveals a trending structural and contractile decline, in the absence of any changes in the expression levels of sMyBP-C or the DRX/SRX ratio of myosin heads. Conversely, 12-month-old E248K female TA muscle shows no signs of structural or contractile deficits, secondary to an upregulation of sMyBP-C expression at 6 months — possibly an early adaptive response — that reverts to downregulation at 12 months, and an accompanied increase of myosin heads in the DRX state. The apparent lack of structural deficits in E248K female TA muscle at 12 months, albeit the decreased levels of sMyBP-C, suggests that there is a critical amount of sMyBP-C that is sufficient to maintain sarcomeric organization. Consistent with this notion, heterozygous carriers of the *MYBPC1* NM_002465.3 c.952C>T (p.R318*) nonsense mutation, residing in the NH_2_-terminal Ig C2 domain and resulting in partial loss of sMyBP-C, are asymptomatic, while homozygous carriers develop Lethal Congenital Contractural Syndrome-4 (LCCS-4), exhibiting perinatal lethality (33). Considering the reported role of sMyBP-C in stabilizing the energetically less demanding SRX state of myosin heads (22, 34), it is tempting to speculate that the reduced levels of sMyBP-C in KI female TA muscles at 12 months may underlie the observed upregulation of myosin heads in the DRX state, possibly serving as an adaptive response, to slow contractile decline. Relatedly, upregulation of the DRX myosin state was recently reported in nemaline myopathy, possibly as an early compensatory mechanism (35). Thus, although at a gross level, Myotrem myopathy manifests similarly in the 2 sexes in mid-adulthood, distinct alterations appear to take place at the biochemical and cellular levels, likely affecting the progression of the disease in late adulthood.

Our intriguing findings raise a major question: what is the basis of the sex-dimorphic and muscle-dependent manifestations of E248K Myotrem (and possibly Myotrem in general) in response to aging? Regarding the observed sex differences in TA contractile function, we suspect that the highly beneficial effects of estrogen in skeletal muscle structure/function, which have been extensively documented (36), may play a role. At the myofilament level, estrogen modulates myosin relaxation kinetics (37, 38) and, thus, actomyosin contractility (22). Accordingly, estrogen depletion via ovariectomy diminishes the lifetime of SRX myosin heads (37), while estrogen attrition due to aging shortens the lifetime of both SRX and DRX myosin heads (38). At the tissue level, estrogen contributes to muscle mass maintenance (39), improves muscle tone and strength (40, 41), and enhances muscle regeneration and repair in response to injury via satellite cell activation (36, 42). These estrogen-mediated protective effects largely vanish once females enter menopause, which is consistent with the unmasking of contractile deficits in the female E248K Myotrem mice in late adulthood, albeit in a muscle-specific manner. While sex and age affect Myotrem manifestation in TA muscle, soleus and gastrocnemius muscles escape such an influence, as they display consistent impairment and preservation, respectively, through mid- and late adulthood regardless of sex.

Of note, while contractile function of soleus muscle was evaluated ex vivo, thereby measuring muscle function in isolation, TA and gastrocnemius muscles were tested in vivo. Here, contractions were evoked via nerve stimulation allowing for capturing total neuromuscular function. Given that patients with Myotrem show no evidence of neuropathy and sMyBP-C expression is restricted to skeletal muscle, the decline in force generation (seen in aged male TA muscle similar to isolated soleus muscle) is likely reflective of intrinsic deficits of muscle architecture, congruent with our quantitative imaging analysis, rather than neural deficits.

The intricacies of individual muscles with regard to their metabolic status (oxidative/slow-twitch versus glycolytic/fast-twitch), mass and cross-sectional area (bigger versus smaller), location in the body (distal versus proximal, axial versus appendicular), and usage (postural versus walking) need to be carefully considered and interrogated, as we unravel Myotrem myopathy. While soleus, gastrocnemius, and TA are distal leg muscles responsible for flexion of the ankle, soleus is unique as it is a relatively small, slow-twitch, tonic muscle (43). Relatedly, these muscles exhibit differential activation patterns during posture (44), gait (45), and motor tasks (46). Accordingly, soleus muscle displays the closest relationship between alterations in muscle length and the center of gravity during proprioception of normal standing, with the TA and gastrocnemius muscles following in correlation strength (44). Moreover, it has been well documented that different muscles exhibit variable susceptibility to distinct clinical pathologies (47, 48). Imaging studies of patients with X-linked myotubular myopathy reveal considerable impairment of soleus muscle (47), while anterior muscles, including the TA, are modestly affected, and posterior muscles, such as the gastrocnemius, are largely spared (47). Similarly, *NEB* mutations that lead to core-rod myopathy typically affect the soleus and TA muscles, while gastrocnemius muscle remains relatively unaffected (49, 50). Conversely, given the distinct levels, combinations, and ratios of sMyBP-C variants across different muscles (3), it is plausible that their differential expression may also contribute to the disparate responses of soleus, gastrocnemius, and TA muscles to Myotrem variants during aging. Thus, a complex and multifaceted interplay between biological sex, aging, and the inherent properties of individual muscle types may underlie the differential progression and manifestation of E248K Myotrem myopathy.

Given the recent recognition of Myotrem, the lack of effective therapies is a major unmet clinical need. As such, patients are often treated with steroids aiming to improve muscle mass and strength, though they experience serious side effects, and β-blockers, commonly used to treat abnormal heart rhythm and hypertension, or primidone, typically used to control epileptic seizures, which however fail to alleviate tremors. Although life expectancy appears not to be affected in E248K Myotrem carriers, skeletal muscle quality and health in late adulthood is of paramount importance when considering factors such as independent walking, physical activity, agility, fall risk, fatigue, and thus overall well-being (51, 52). The surge in reported cases of Myotrem (15–18, 29) since its original identification in 2019 (9, 10), coupled with its nonprogressive phenotype from adolescence through mid-adulthood, may imply that the actual prevalence of Myotrem is higher than what was originally presumed. Thus, deciphering the disease pathogenesis and presentation as a function of biological sex, muscle type, and aging is imperative for the comprehensive characterization of the disease and the design of effective and targeted therapies.

## Methods

### Sex as a biological variable.

Our study examined male and female animals, and sex-dimorphic effects are reported.

### Survival curves.

Kaplan-Meier curves were generated for WT and heterozygous KI animals from 4 weeks through 24 months of age. Statistical significance comparing survival rates between WT and KI was performed using the log-rank test.

### Inverted hang assay.

Mice were placed on a wire grid hung ~50 cm above a soft padded surface. The grid was slowly inverted over a time period of 2 seconds. Hang time was measured as the time from full inversion to until the animal dropped. If the mouse did not fall after 3 minutes, the assay was stopped, and the maximum time of 180 seconds was recorded. The animal was returned to its cage and allowed to rest for at least 3 minutes. The assay was repeated 3 times in 1 day. Individual data points reflect the average of these 3 trials per animal.

### DEXA.

Animals were lightly anesthetized and exposed to DEXA (iNSiGHT VET DXA, Osteosys) scans. For body composition measurements, the animals were placed in a prone position during the scan. A region of interest (ROI) of the animal body excluding the head and tail was used for estimations of total mass, lean mass, and fat mass. For spinal curvature measurements, the animal was placed in a right lateral recumbent position during the scan. Kyphotic index was determined as previously described (12, 53) by calculating the ratio of the distance between the C7 and L6 vertebrae and the length of a perpendicular line reaching the extreme dorsal curvature of the spine.

### Ex vivo assessment of soleus contractile function.

In vaporized isoflurane-anesthetized mice, the soleus was secured with sutures at each tendon, immersed in an ex vivo temperature-controlled (25°C) perfusion bath of oxygenated (95% O_2_, 5% CO_2_) Ringer’s solution (140 mM NaCl, 4 mM KCl, 1 mM MgSO_4_, 10 mM HEPES, 10 mM glucose, 5 mM NaHCO_3_, 1.8 mM CaCl_2_, pH 7.4), and mounted to a length controller and force transducer (1200A, Aurora Scientific) at optimal length. Optimal length (L_0_) was determined by iteratively adjusting muscle length to achieve maximal twitch (1 Hz) force. The force-frequency relationship was determined by delivering brief (500 ms) trains of field pulses of 1–200 Hz delivered by plate electrodes parallel to each side of the muscle. The resulting force of isometric contraction was measured at a sampling rate of 10,000 Hz. PCSA was calculating using the equation, 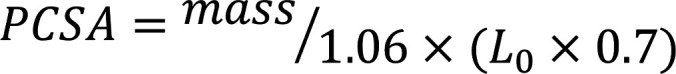 where mass is the isolated soleus mass and L_0_ is optimal length. Soleus specific force was calculated by dividing the absolute peak force by the PCSA. Measurements from tetanic stimulation (200 Hz) were used as outcome variables.

### In vivo assessment of gastrocnemius and TA function.

In vaporized isoflurane-anesthetized mice, the hindlimb was immobilized at the knee and the foot was secured to a force transducer (1300A, Aurora Scientific). The tibial nerve was then stimulated with brief (500 msec) trains of pulses delivered at 1–150 Hz to elicit isometric contractions of the gastrocnemius muscle, and the resulting force was measured. The protocol was repeated, stimulating the common peroneal nerve to measure contraction of the TA muscle. The data were plotted to display the prototypic force-frequency relationship, and the tetanic measurements delivered at 150 Hz were used as outcome variables. Specific force was calculated by dividing the absolute peak force measurement by the mass of the isolated muscle.

### Generation of protein lysates and immunoblotting.

Freshly isolated TA muscles were harvested from mice using flash-frozen liquid nitrogen. A small (~15 mg) portion of the mid-belly of the muscle was sampled for lysate generation via hand-homogenization in a modified NP-40 lysis buffer (10 mM NaH_2_PO_4_ [pH 7.2], 2 mM EDTA, 10 mM NaN_3_, 120 mM NaCl, 0.5% deoxycholate, 1% NP-40) supplemented with cOmplete protease inhibitor (Roche) and Halt phosphatase inhibitor (Thermo Fisher Scientific) cocktails. NuPage LDS sample buffer and reducing agent (Invitrogen) were added to 20 μg of protein lysates, boiled at 95°C for 5 minutes, and fractionated by 4%–12% SDS-PAGE. Protein was transferred to nitrocellulose membrane and blocked with 5% milk (RPI) in tris buffered saline containing 0.1% Tween 20. Blot strips were then probed with antibodies against sMyBP-C (rabbit polyclonal, SAB3501005, 1:12,000, MilliporeSigma) or GAPDH (mouse monoclonal, G8795, 1:30,000, MilliporeSigma) at 4°C overnight. Blots were subsequently incubated in the appropriate horseradish peroxidase–conjugated secondary antibody (Cell Signaling Technology) and ECL substrate (Thermo Fisher Scientific). Densitometry was performed with ImageJ software (NIH), and the average value of 2 technical replicates was used for analysis.

### Single nucleotide turnover Mant-ATP assay.

Biochemical assessment of the myosin relaxation states was determined using single nucleotide turnover Mant-ATP chase experiments, as previously reported (35). In short, mice were humanely euthanized via CO_2_ inhalation, followed by cervical dislocation. The TA muscle was quickly harvested and placed in a skinning solution of 1:1 glycerol to relaxing solution containing 4 mM Mg-ATP, 1 mM free Mg^2+^, 1 × 10^–6^ mM free Ca^2+^, 20 mM imidazole, 7 mM EGTA, 14.5 mM creatine phosphate, and sufficient KCl to adjust the ionic strength to 180 mM and the pH to 7.0. The tissue was pinned on Sylgard plates to maintain its resting length, incubated in relaxing solution at –20°C for 48 hours, and subsequently cryopreserved at –80°C until experimentation.

On the day of experimentation, bundles were thawed and single myofibers were mechanically isolated and mounted inside a flow chamber at a resting sarcomere length of ~2.20 μm. Fibers were incubated with rigor buffer, containing 120 mM K acetate, 5 mM Mg acetate, 2.5 mM K_2_HPO_4_, 50 mM MOPS, 2 mM DTT (pH 6.5), for 5 minutes. The flow chamber was then flushed with rigor buffer containing 250 μM Mant-ATP and allowed to incubate for 5 minutes. To complete the chase, the flow chamber was subsequently flushed with rigor buffer containing 4 mM unlabeled ATP. Florescence was acquired using a Zeiss Axio Scope A1 microscope, and 3 ROIs per myofiber were used for analysis. Normalized florescence intensity signals were fit to an unconstrained double exponential decay curve using GraphPad Prism (GraphPad Software): 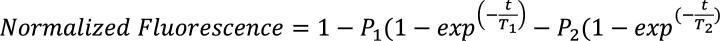 where the initial rapid decay corresponds to the DRX state and the slower second decay corresponds to the SRX state of myosin heads. P_1_ and P_2_ represent the respective amplitudes, while T_1_ and T_2_ denote the respective time constants.

### Immunofluorescence combined with confocal microscopy and image processing.

Mice were deeply anesthetized and perfused with 4% paraformaldehyde in PBS. The TA muscle was rapidly harvested and embedded in Tissue-Tek OCT Compound (Sakura). Cryosections (10 μm thick) were cut, permeabilized with 0.1% Triton-X in PBS for 20 minutes, and blocked with 2% goat serum, 2% BSA, and 1 mM NaN_3_ in PBS for 30 minutes at room temperature. Tissue sections were labeled with primary antibodies against sarcomeric α-actinin (mouse monoclonal, A7811, 1:1,000, MilliporeSigma) and sMyBP-C (rabbit polyclonal, SAB3501005, 1:250, MilliporeSigma). Samples were counterstained with the appropriate fluorophore-conjugated secondary antibody (Alexa Fluor 488 or Alexa Fluor 568, A11034 or A21043, respectively; 1:250, Thermo Fisher Scientific) along with Hoechst 33258 dye (Thermo Fisher Scientific) and subsequently mounted with ProLong Diamond Antifade Mountant (Thermo Fisher Scientific). Slides were imaged using a Nikon Ti2 inverted spinning disk confocal microscope, and images were taken at 60× magnification. Five representative ROIs from a total of 3 biological samples per sex/per genotype/per age were analyzed. Images were compiled into a *z*-stack, processed, and quantitatively analyzed for sarcomeric structural organization and sMyBP-C localization using FIJI software (NIH).

### Quantification of sarcomeric organization and sMyBP-C localization.

Tissue sections (Supplemental Figure 11, A and D) were coimmunostained for α-actinin and sMyBP-C. α-Actinin–stained images (Supplemental Figure 11, B and E) were used to calculate sarcomeric breakage, continuity, and order scores. In particular, images underwent skeletonization and branching analysis using the Skeletonize3D and AnalyzeSkeleton FIJI plugins. The number and length of branches were used to calculate the “breakage” and “continuity” scores, respectively. Moreover, α-actinin–stained images were subjected to Fast Fourier Transform (FFT) analysis (Supplemental Figure 11, G and I) to determine the sarcomeric “order” score based on the regularity of Z-disk periodicity, as previously described (54). The resulting power spectra were analyzed using a customized Python script (https://github.com/humbertojoca/FFT_peaks; commit ID bcf80aab0ea5dd5b8d57c31046a0a40cb6a6b37b) that calculated the relationship of intensity versus spatial frequency. The first peak, located at ~0.4 μm^–1^ on the power spectrum (Supplemental Figure 11, G and I; green arrow), and the corresponding peak in the resulting trace (Supplemental Figure 11, K and M; marked in green), reflect sarcomeric “order.” The amplitude of the first peak was reported as the “order” score.

The breakage, continuity, and order scores were then collectively used to classify the type of sarcomeric (dis)organization shown in Supplemental Figure 12, as: prototypic sarcomeric organization, characterized by a regular striated pattern (Supplemental Figure 12A); lateral misalignment, characterized by lateral dissociation of adjacent myofibrils (Supplemental Figure 12B); sarcomeric continuum, characterized by uninterrupted wavy-like striations (Supplemental Figure 12C); disordered organization, characterized by irregular and out-of-register sarcomeres in the absence of lateral breakage (Supplemental Figure 12D); and chaotic organization, characterized by irregular and out-of-register sarcomeres in the presence of lateral breakage (Supplemental Figure 12E).

The localization of sMyBP-C to the C-zone in its characteristic doublet was evaluated using 2 methods. The first method qualitatively evaluated sMyBP-C distribution along a ~10 μm ROI using FIJI. Specifically, normalized signal intensity values of α-actinin (marker of overall sarcomeric structure) and sMyBP-C were plotted versus distance along the ROI to visualize their striated localization patterns. The second method quantitatively evaluated sMyBP-C localization throughout the entire image. sMyBP-C–stained images (Supplemental Figure 11, C and F) underwent FFT analysis (Supplemental Figure 11, H and J), as described in ref. 54. The resulting power spectra were analyzed using a customized Python script (https://github.com/humbertojoca/FFT_peaks; commit ID bcf80aab0ea5dd5b8d57c31046a0a40cb6a6b37b), and the intensity versus spatial frequency was plotted. The second peak, located at ~0.8 μm^–1^ on the power spectrum (Supplemental Figure 11 H and J; orange arrow), and the corresponding trace (Supplemental Figure 11, L and N) reflect sMyBP-C localization to the C-zone. The amplitude of the second peak (Supplemental Figure 11, L and N) was reported as the sMyBP-C “localization” score.

### Statistics.

Statistical tests, sample sizes (*n*), and *P* values are provided in figure legends. Data are presented as mean ± SEM and were analyzed by GraphPad Prism. The D’Agostino & Pearson test was used to examine normality, and an F test was used to compare variances. Comparisons between 2 normal datasets of similar variance were performed using Student’s 2-tailed *t* test. Mann-Whitney *U* test or Welch’s 2-tailed *t* test was used for datasets that failed the normality or variance test, respectively, and a *P* value less than 0.05 was considered significant. Values are expressed as mean ± SEM.

### Study approval.

All animal work was conducted under protocols approved by the IACUC of the University of Maryland Baltimore. Studies use 6-, 12-, and 24-month-old WT and heterozygous E248K KI C57BL/6J mice from the same breeding scheme. Generation of the KI murine model was previously described in ref. 12 and contains the murine *Mybpc1* E249K substitution corresponding to the human E248K variant. KI mice are referred to as the *MYBPC1* E248K KI murine model to avoid confusion. The model is maintained with regular backcrossing to avoid genetic drifting and off-target effects.

### Data availability.

Original blots and images as well as raw data values are included in the supplemental materials and Supporting Data Values files.

## Author contributions

Conceptualization was contributed by JMM, CW, and AKK. Data curation was contributed by JMM, HCJ, JK, NR, JO, and CW. Formal analysis was contributed by JMM, HCJ, JK, NR, JO, and CW. Funding acquisition was contributed by JO, CW, and AKK. Investigation was contributed by JMM, HCJ, JK, NR, JO, CW, and AKK. Methodology was contributed by JMM, HCJ, and CW. Project administration was contributed by JO, CW, and AKK. Resources were contributed by JO, CW, and AKK. Software was contributed by HCJ. Supervision was contributed by CW and AKK. Validation was contributed by JMM, HCJ, JK, JO, CW, and AKK. Visualization was contributed by JMM and HCJ. The writing of the original draft was contributed by JMM. Reviewing and editing of the manuscript was contributed by JMM, HCJ, JK, NR, JO, CW, and AKK.

## Supplementary Material

Supplemental data

Unedited blot and gel images

Supplemental video 1

Supplemental video 2

Supplemental video 3

Supplemental video 4

Supplemental video 5

Supplemental video 6

Supplemental video 7

Supplemental video 8

Supporting data values

## Version 1 06/26/2025

In-Press Preview

## Version 2 08/08/2025

Electronic publication
