# Supplementary material for: Sex-dependent and muscle-specific progression of the MYBPC1 E248K Myotrem myopathy in response to aging
Source: JCI Insight. 2025 Jun 26;10(15):e182471. doi: 10.1172/jci.insight.182471 (PMC12341543; doi:10.1172/jci.insight.182471)
Supplement: Supplemental data [file jciinsight-10-182471-s023.pdf]

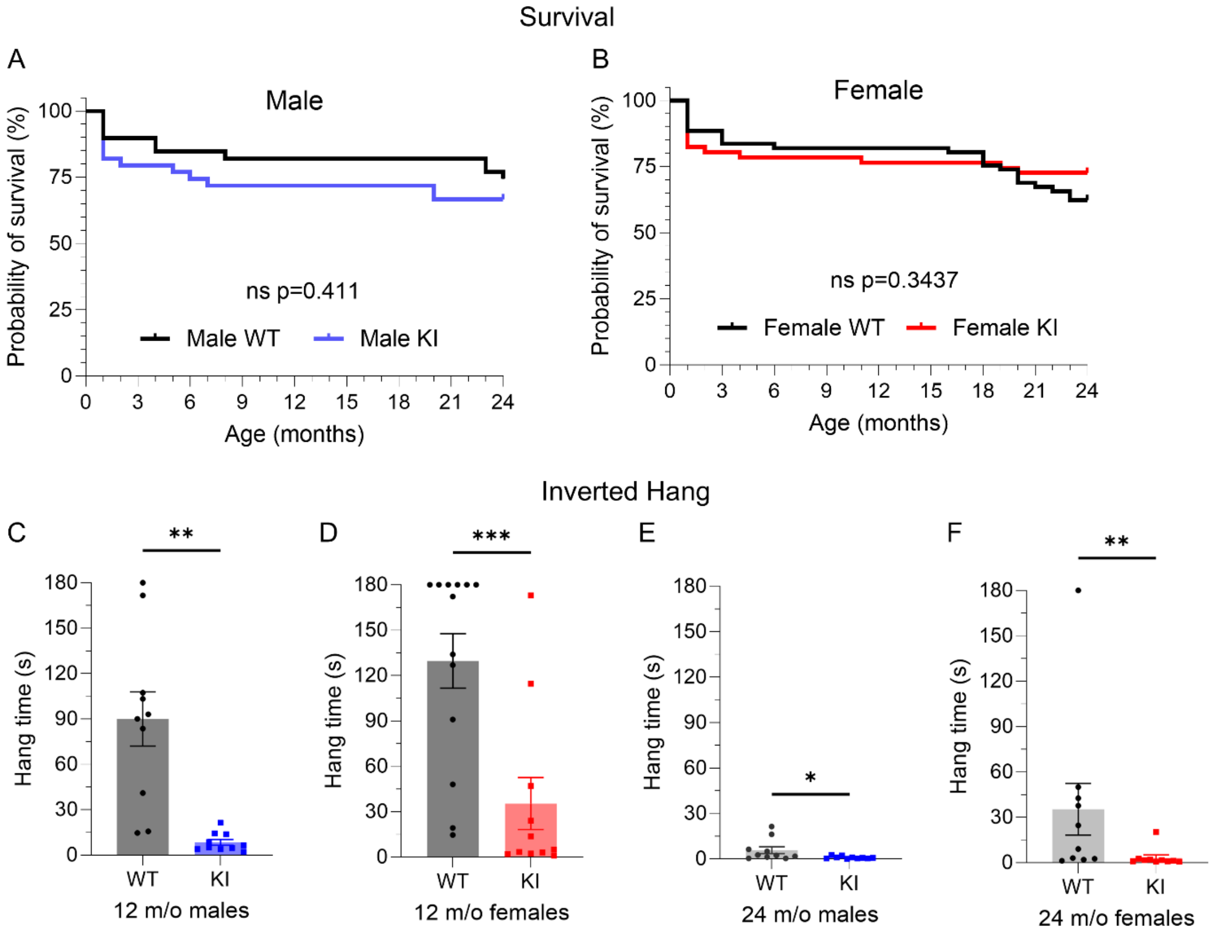

Supplemental Figure 1. Survival and neuromuscular performance evaluation of E248K KI Myotrem mice in mid- and late adulthood. (A and B) Kaplan-Maier curves show no difference in survival rates between WT and Knock-In (KI) animals, independently of biological sex, between 4-weeks to 24-months of age; log-rank test, males: p=0.603, n=39 per genotype; females: p=0.344, n=59 WT, n=51 KI. **(C-F)** Overall coordination, endurance, and neuromuscular performance were assessed by inverted hang assay. KI animals consistently performed worse than their age-and sex-matched WT littermates; 12-month-old males: n=10 per genotype, 24-month-old males: n=10 per genotype, 12-month-old females: n=13 WT, n=11 KI; 24-month-old females: n=10 per genotype. Data is presented as mean ± SEM and statistical significance was determined by Welch's t-test (C) and Mann-Whitney test (D-F); *$p < 0.05$, **$p < 0.01$, and ***$p < 0.001$.

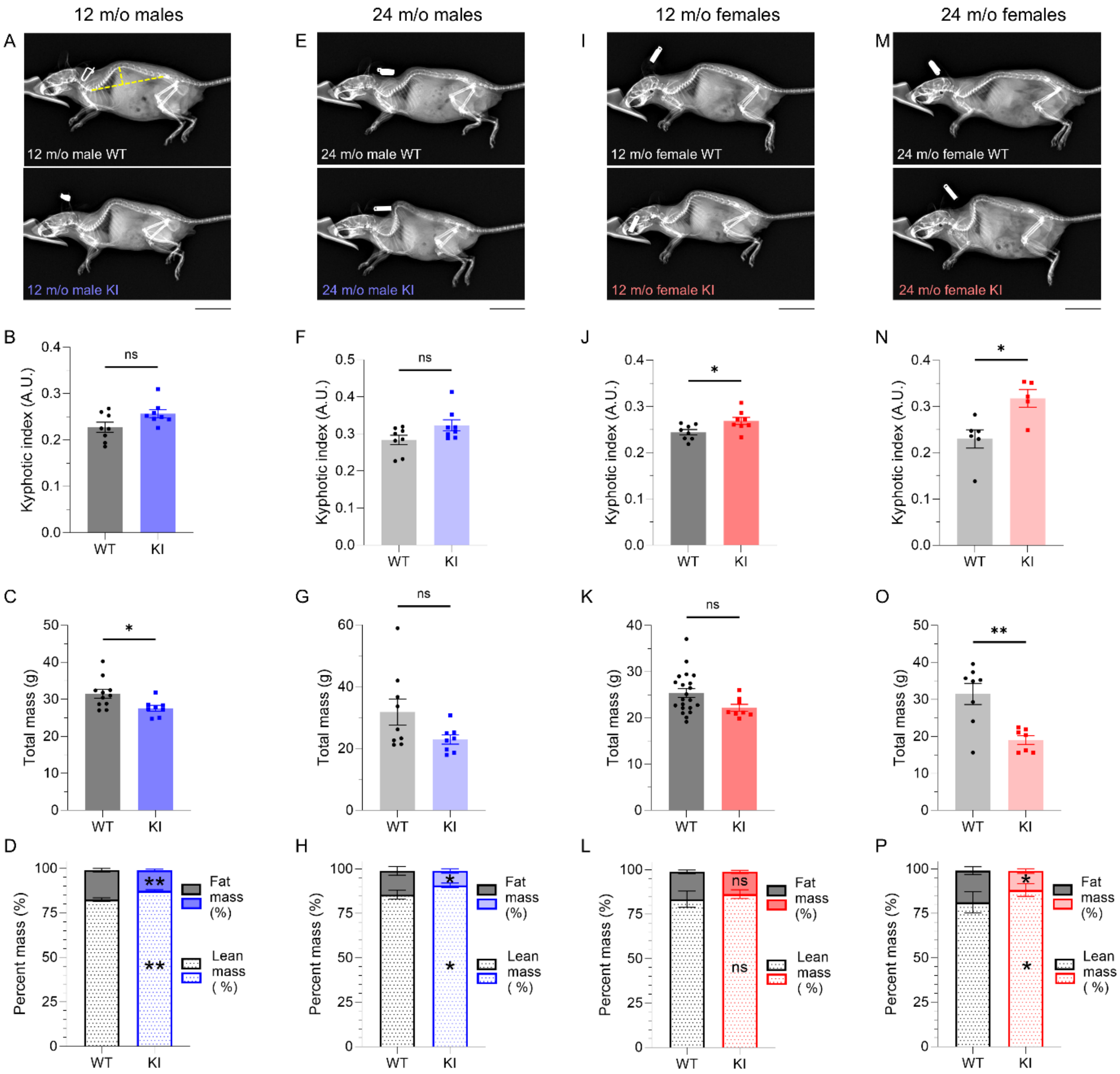

Supplemental Figure 2. Phenotypic evaluation of aging E248K Myotrem mice using DEXA. Dual-energy X-ray absorptiometry (DEXA) measurements were performed in 12- **(A-D)** and 24-month-old **(E-H)** WT and Knock-In (KI) males as well as 12- **(I-L)** and 24-month-old **(M-O)** WT and KI females. **(A, E, I, and M):** Representative DEXA scans show body and skeletal morphology; scale bar: 2 cm. **(B, F, J, and N):** The extent of spinal curvature is quantified by the kyphotic index calculated as the ratio of the distance between the C7 and L6 vertebrae and the length of a perpendicular line reaching the extreme dorsal curvature of the spine, indicated by the dotted yellow line in the WT animal shown in **(A).** Kyphotic index trends to be higher in 12- and 24-month-old KI males **(B and F),** and is significantly elevated in 12- and 24-month-old KI females **(J and N)**, when compared to age- and sex-matched WT; *n*=8 males per age/per genotype, *n*=5

12-month-old females per genotype, *n*=6 24-month-old WT females, and *n*=5 24-month-old KI females. **(C, G, K, and O):** Total mass measurements indicated that KI animals have either significantly (i.e., 12-month-old males and 24-month-old females) or trending (12-month-old females and 24-month-old males) smaller mass compared to their age- and sex-matched WT littermates. **(D, H, L, and P):** KI males are consistently leaner than their age-matched WT counterparts at both 12-months **(D)** and 24-months **(H)**. While WT and KI females exhibit comparable percentages or relative lean mass composition at 12-months **(L)**, by 24-months, KI mice are markedly leaner than their age- and sex-matched WT littermates **(P)**; *n*=11 12-month-old WT males, *n*=8 12-month-old KI males, *n*=9 24-month-old WT males, *n*=8 24-month-old KI males, *n*=21 12-month-old WT females, *n*=8 12-month-old KI females, *n*=8 24-month-old WT females, and *n*=7 24-month-old KI females. Data is presented as mean ± SEM and statistical significance was determined by 2-tailed Student's *t*-test (B-D, J-K, N and P), Welch's *t*-test, (G and O), and Mann-Whitney test (F, H, and L); ns: not significant, *$p < 0.05$ and **$p < 0.01$.

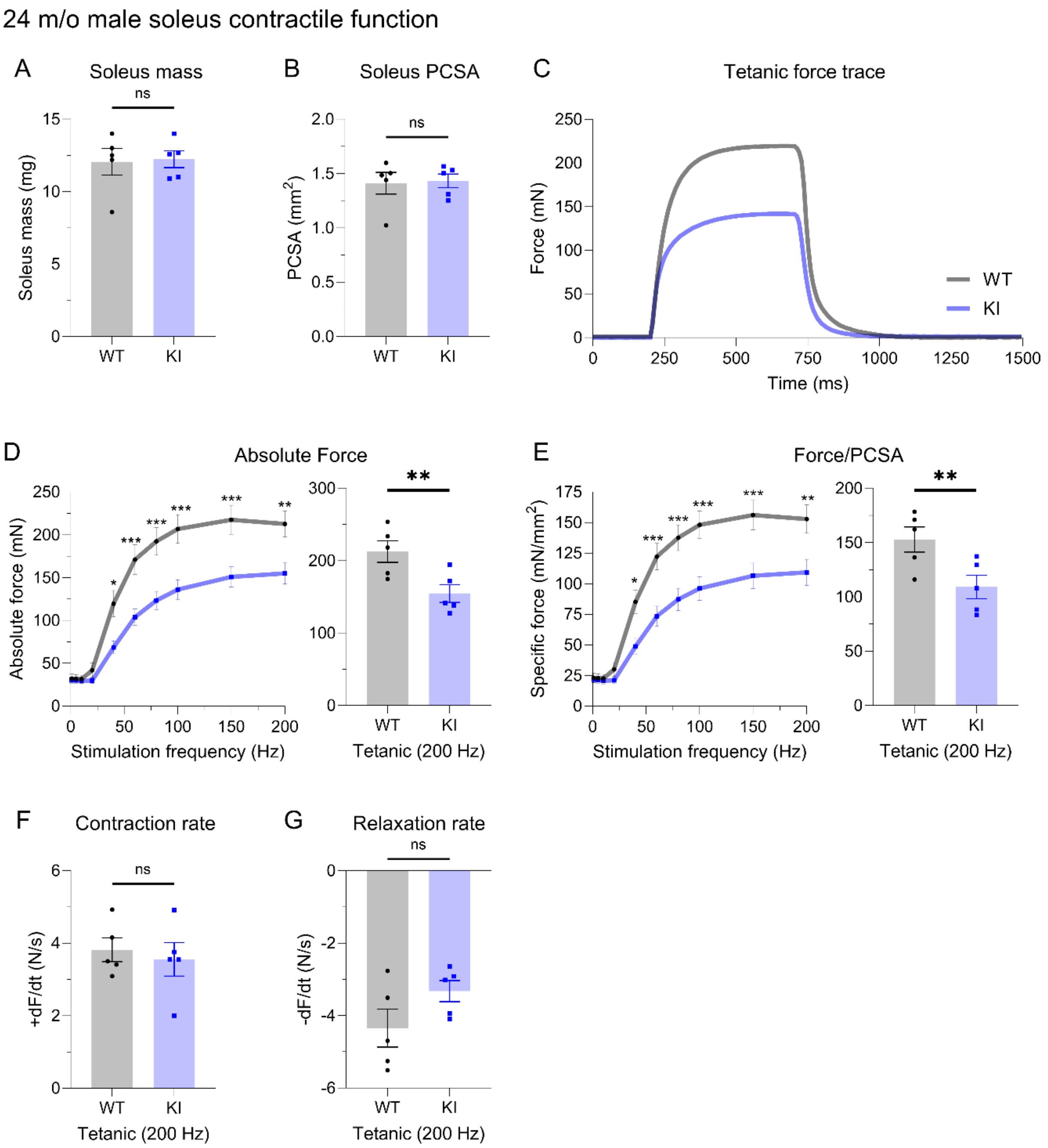

Supplemental Figure 3. Ex vivo contractile function of 24-month-old male soleus muscle. The force-frequency relationship of soleus muscle from WT and Knock-In (KI) 24-month-old male mice was evaluated using an ex vivo system. Isolated soleus mass **(A)** and physiological cross-sectional area (PSCA; **B)** were comparable between the two genotypes. Analysis of contractile function following tetanic stimulation **(C)** demonstrated impaired absolute **(D)** and specific **(E)** force in KI soleus relatively to age- and sex-matched WT. However, contraction **(F)** and relaxation **(G)** kinetics were similar between genotypes; *n*=5 male mice per genotype. Data is presented as

mean ± SEM and force traces are shown over a 1500 msec period with a sampling rate of 125 Hz. Statistical significance was determined by 2-tailed Student's *t*-test (A-B and F-G) and 2-way ANOVA followed by Šídák's multiple comparisons test (D and E); ns: not significant, *$p < 0.05$, **$p < 0.01$, and ***$p < 0.001$.

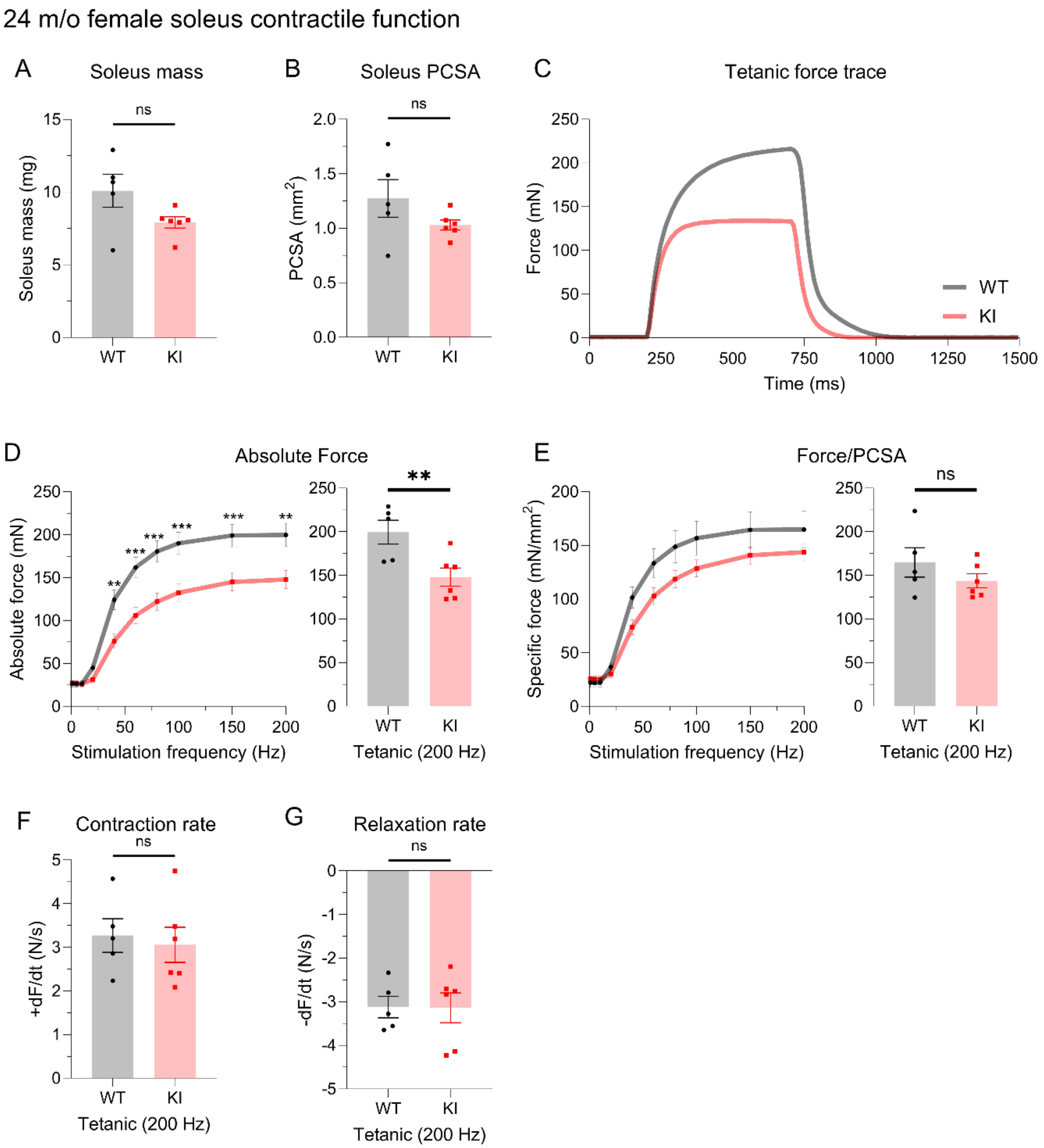

Supplemental Figure 4. Ex vivo contractile function of 24-month-old female soleus muscle. Differences in soleus mass **(A)** and physiological cross-sectional area (PSCA; **B)** between WT and Knock-In (KI) soleus from 24-month-old female mice did not reach statistical significance. The force frequency relationship was plotted **(C)** and values of force production after tetany and contractility kinetics were used as output variables. **(D)** KI solei produced lower absolute force compared to WT. **(E)** However, when absolute force was normalized to soleus PSCA, intrinsic

specific force was similar between WT and KI female solei. Measured rates of contraction **(E)** and relaxation **(F)** were also comparable between WT and KI groups; n=5 WT and n=6 KI mice. Data is presented as mean ± SEM and force traces are shown over a 1500 msec period with a sampling rate of 125 Hz. Statistical significance was determined by 2-tailed Student's *t*-test (A, F, and G), Welch's *t*-test, (B), and 2-way ANOVA followed by Šidák's test for multiple comparisons (D and E); ns: not significant, ***p* < 0.01, and ****p* < 0.001.

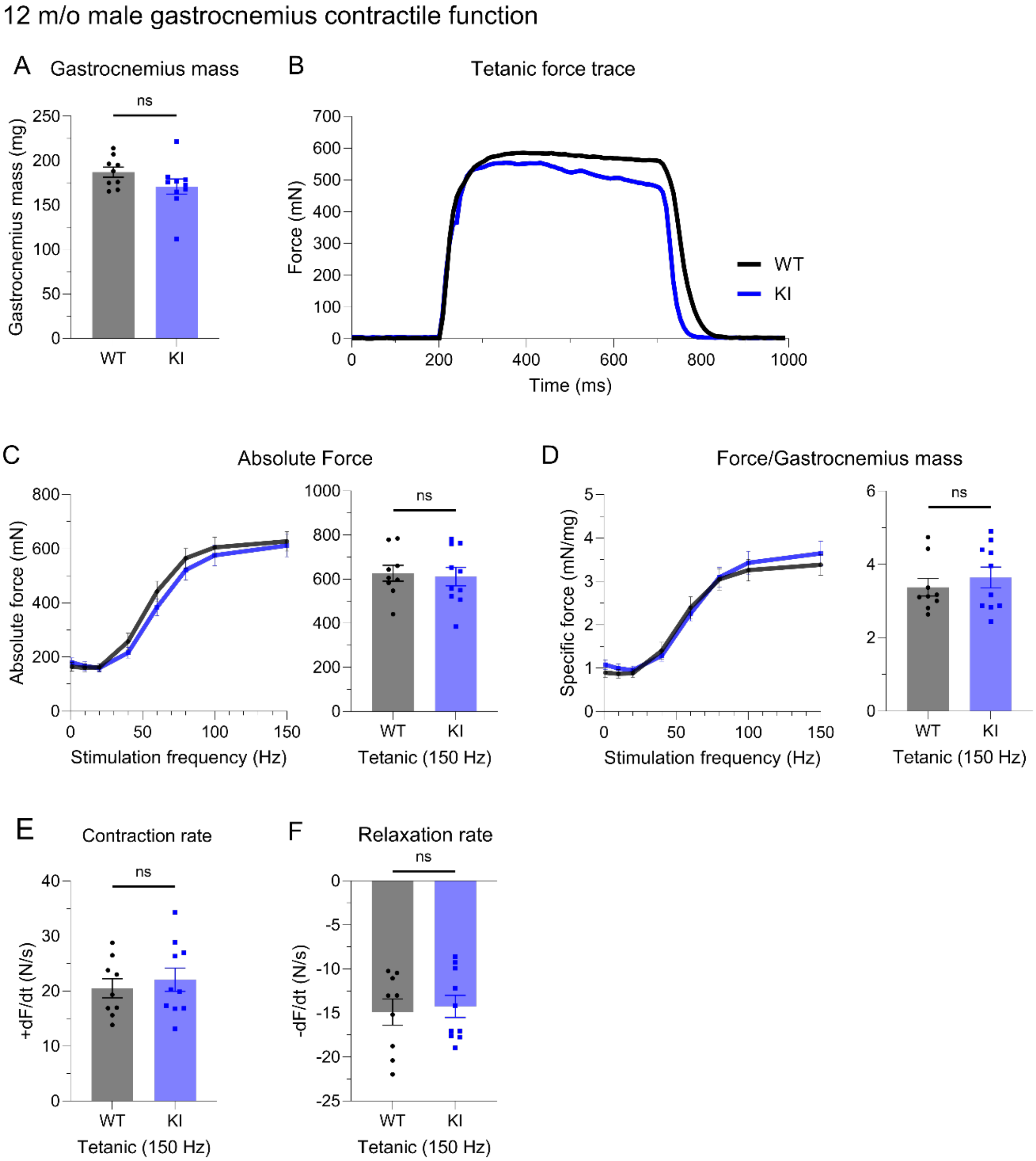

Supplemental Figure 5. In vivo contractile function of 12-month-old male gastrocnemius muscle. Contractility was assessed using in vivo nerve-evoked isometric contractions. WT and Knock-In (KI) mice were anesthetized, the hindlimb was immobilized, and the tibial nerve was percutaneously stimulated by brief (500 msec) trains of pulses delivered at 1 to 150 Hz. **(A)** Isolated gastrocnemius muscle mass was comparable between WT and KI males at 12-months. **(B)** Representative force traces of gastrocnemius plantar flexion of WT and KI 12-month-old mice

resulting from 150 Hz stimulation. **(C, left)** Force *versus* stimulation frequency curve. No differences between WT and KI gastrocnemius muscles were observed in absolute tetanic force **(**150 Hz; **C, right**), specific tetanic force (calculated by dividing the absolute tetanic force with the muscle mass) **(D)**, or the rates of contraction **(E)** and relaxation **(F)** at 12-months; *n*=9 WT and *n*=10 KI mice. Data is presented as mean ± SEM and force traces are shown over a 1000 msec period with a sampling rate of 125 Hz. Statistical significance was determined by 2-tailed Student's *t*-test (A, E, and F) and 2-way ANOVA followed by Šidák's test for multiple comparisons (C and D); ns: not significant.

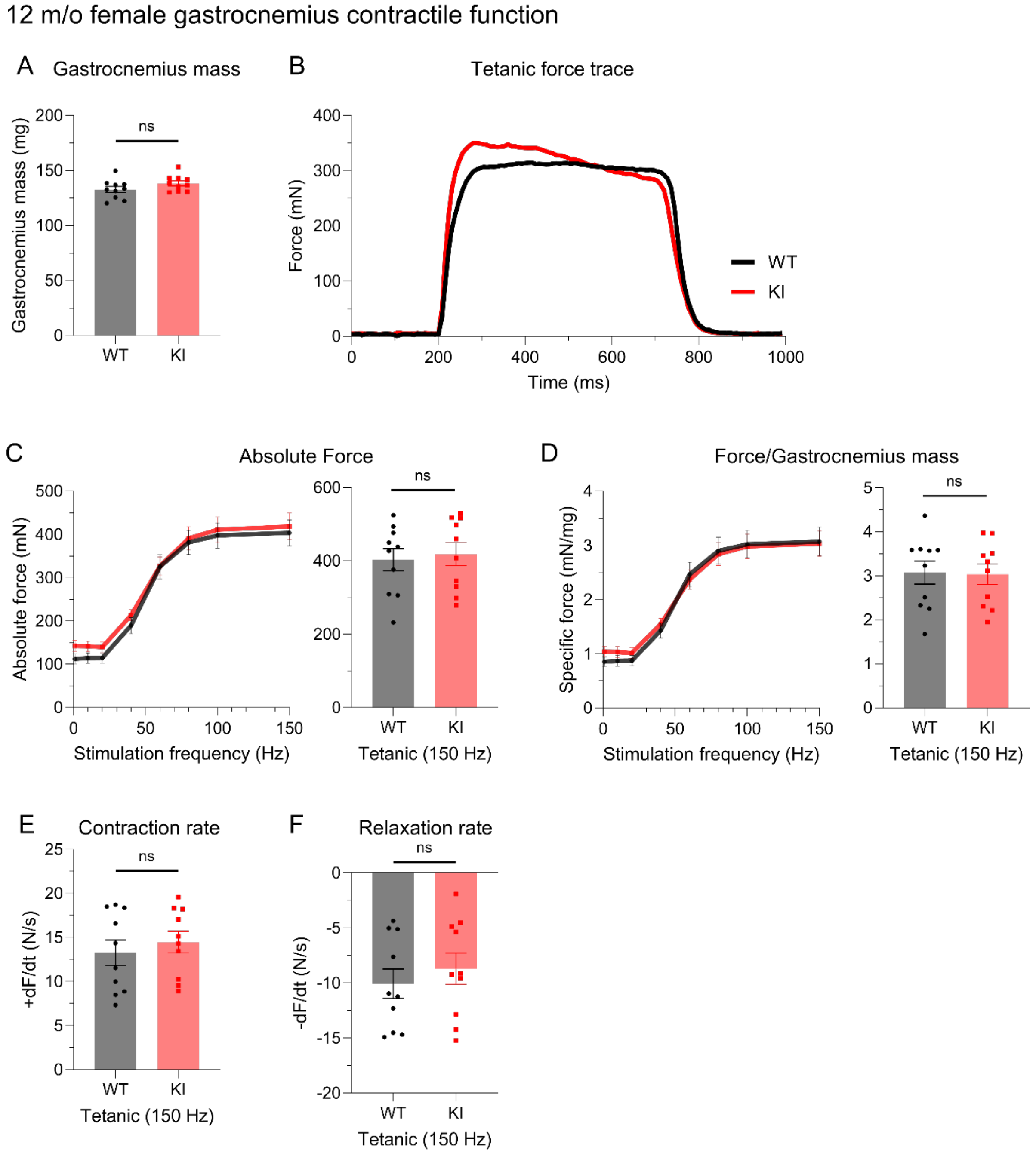

Supplemental Figure 6. In vivo contractile function of 12-month-old female gastrocnemius muscle. Contractility was assessed using in vivo nerve-evoked isometric contractions. The tibial nerve was percutaneously stimulated by brief (500 msec) trains of pulses delivered at 1 to 150 Hz. Knock-In (KI) female gastrocnemius muscles exhibited comparable mass **(A)** and developed similar absolute **(B and C)** and specific **(D)** tetanic force, as well as contraction **(E)** and relaxation **(F)** kinetics to WT; *n*=10 mice per genotype. Data is presented as mean ± SEM and force traces are shown over a 1000 msec period with a sampling rate of 125 Hz. Statistical significance was

determined by 2-tailed Student's *t*-test (A, E, and F) and 2-way ANOVA followed by Šidák's test for multiple comparisons (C and D); ns: not significant.

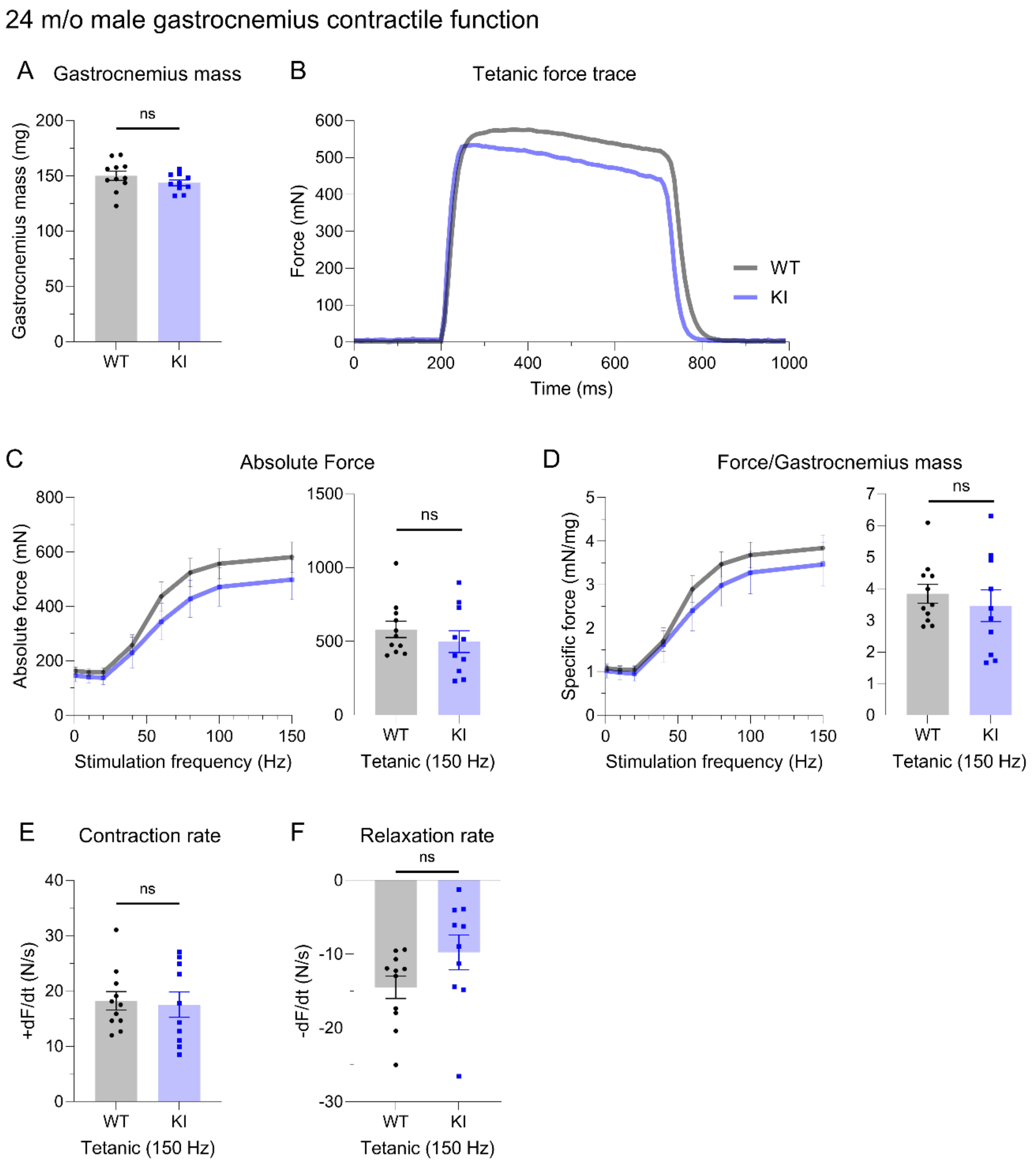

Supplemental Figure 7. In vivo contractile function of 24-month-old male gastrocnemius muscle. Contractility was assessed using in vivo nerve-evoked isometric contractions. The tibial nerve was percutaneously stimulated by brief (500 msec) trains of pulses delivered at 1 to 150 Hz. Muscle mass between WT and Knock-In (KI) gastrocnemius male muscles was comparable at 24-months. Additionally, absolute force **(B and C)**, specific force **(D)**, and the rates of contraction **(E)** and relaxation **(F)** were similar between WT and KI males; *n*=11 WT and *n*=10 KI mice. Data is presented as mean ± SEM and force traces are shown over a 1000 msec period

with a sampling rate of 125 Hz. Statistical significance was determined by 2-tailed Student's *t*-test (A, E, and F) and 2-way ANOVA followed by Šidák's test for multiple comparisons (C and D), as appropriate; ns: not significant.

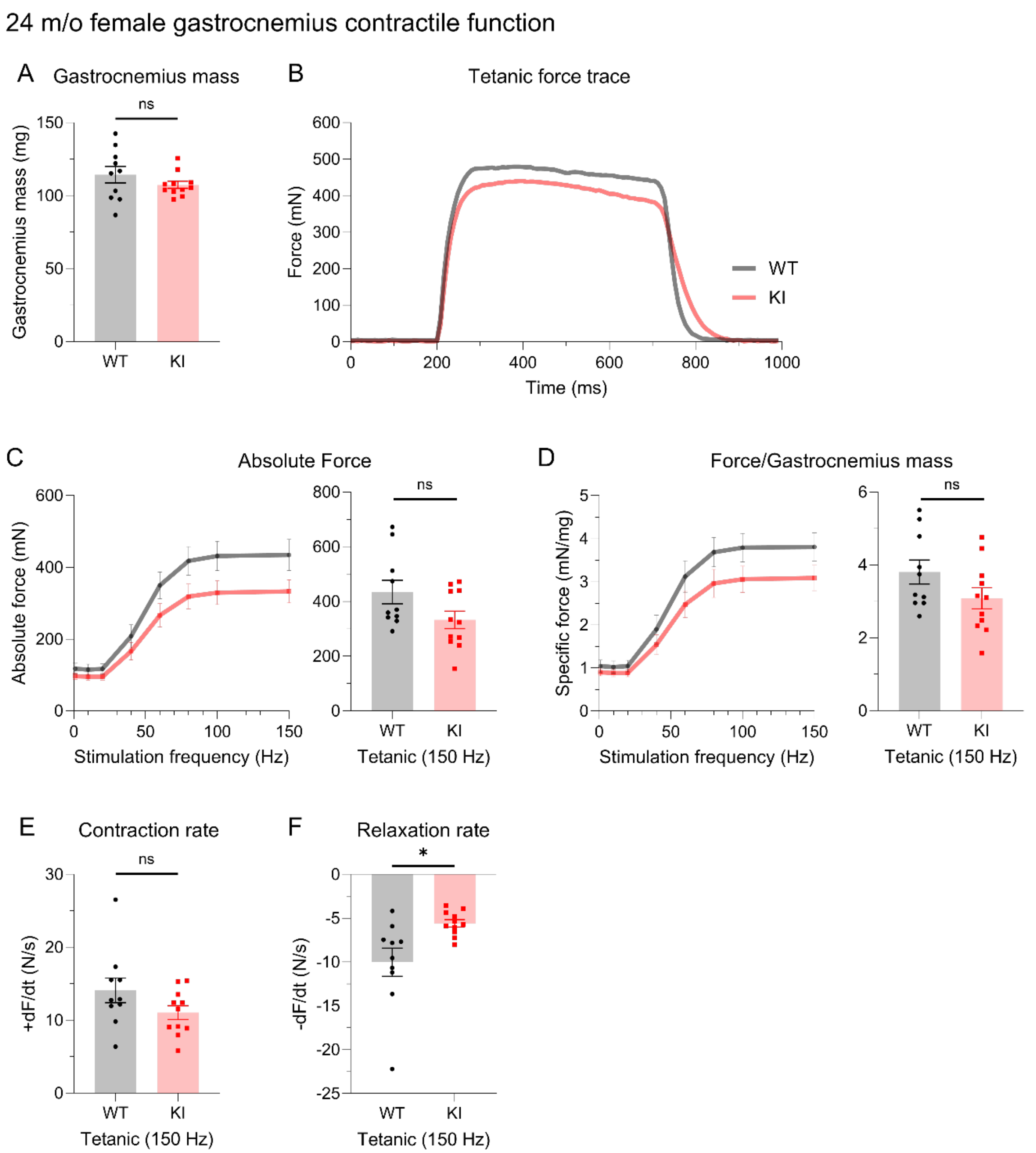

Supplemental Figure 8. In vivo contractile function of 24-month-old female gastrocnemius muscle. Contractility was assessed using in vivo nerve-evoked isometric contractions. The tibial nerve was percutaneously stimulated by brief (500 msec) trains of pulses delivered at 1 to 150 Hz. At 24-months, Knock-In (KI) female gastrocnemius muscles exhibit similar muscle mass **(A)**, absolute force **(B and C)**, specific force **(D)**, and contraction rate **(E)** compared to WT. **(F)** Interestingly though, the relaxation rate exhibited by KI female mice is lower than that of WT at 24-months; *n*=10 WT and *n*=11 KI mice. Data is presented as mean ± SEM and force traces are

shown over a 1000 msec period with a sampling rate of 125 Hz. Statistical significance was determined by 2-tailed Student's *t*-test (E), Welch's *t*-test (A and F) and 2-way ANOVA followed by Šidák's test for multiple comparisons (C and D); ns: not significant and $^{*}p < 0.05$.

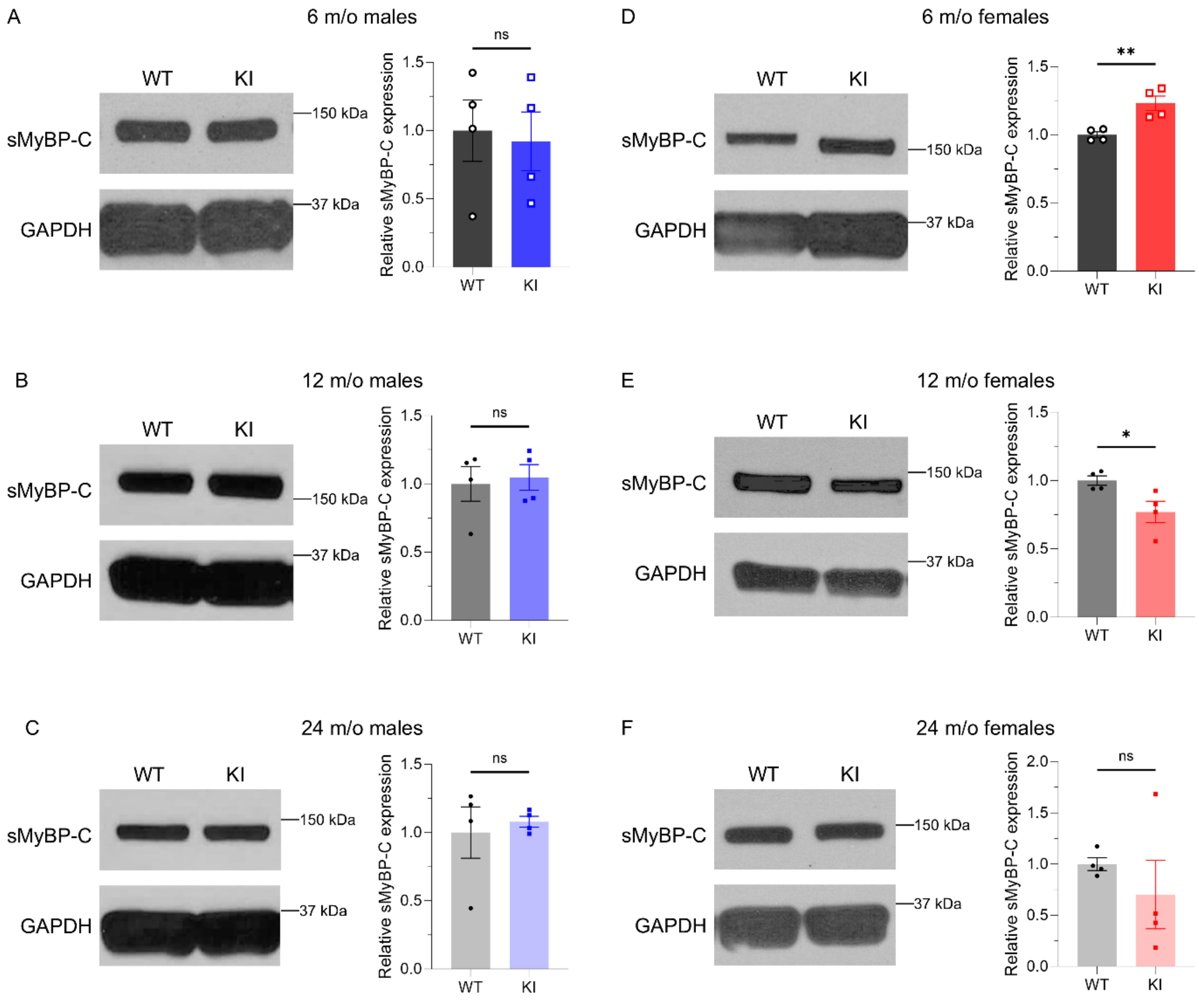

Supplemental Figure 9. Immunoblot analysis of sMyBP-C expression levels in E248K Tibialis Anterior muscle through aging. Representative immunoblots probed for slow Myosin Binding Protein-C (sMyBP-C) levels using lysates prepared from male **(A-C)** and female **(D-F)** Tibialis Anterior (TA) muscles at 6- **(A and D)**, 12- **(B and E)**, and 24-months **(C and F)** of age. Quantification is relative to sex- and age-matched WT. **(A-C)** Male Knock-In (KI) TA muscles express the same levels of sMyBP-C compared to WT at 6-, 12-, and 24-months of age. On the contrary, female KI TA muscles show significantly increased sMyBP-C expression at 6-months **(D)**, which reverts to marked reduction at 12-months **(E),** and remains as a strong decreasing trend at 24-months **(F)**; n=4 mice per sex/per age/per genotype. Data is represented as mean ± SEM and statistical significance was determined by 2-tailed Student's t-test (A, B, D, and E) with Welch's correction (C and F); ns: not significant, $*p < 0.05$ and $**p < 0.01$.

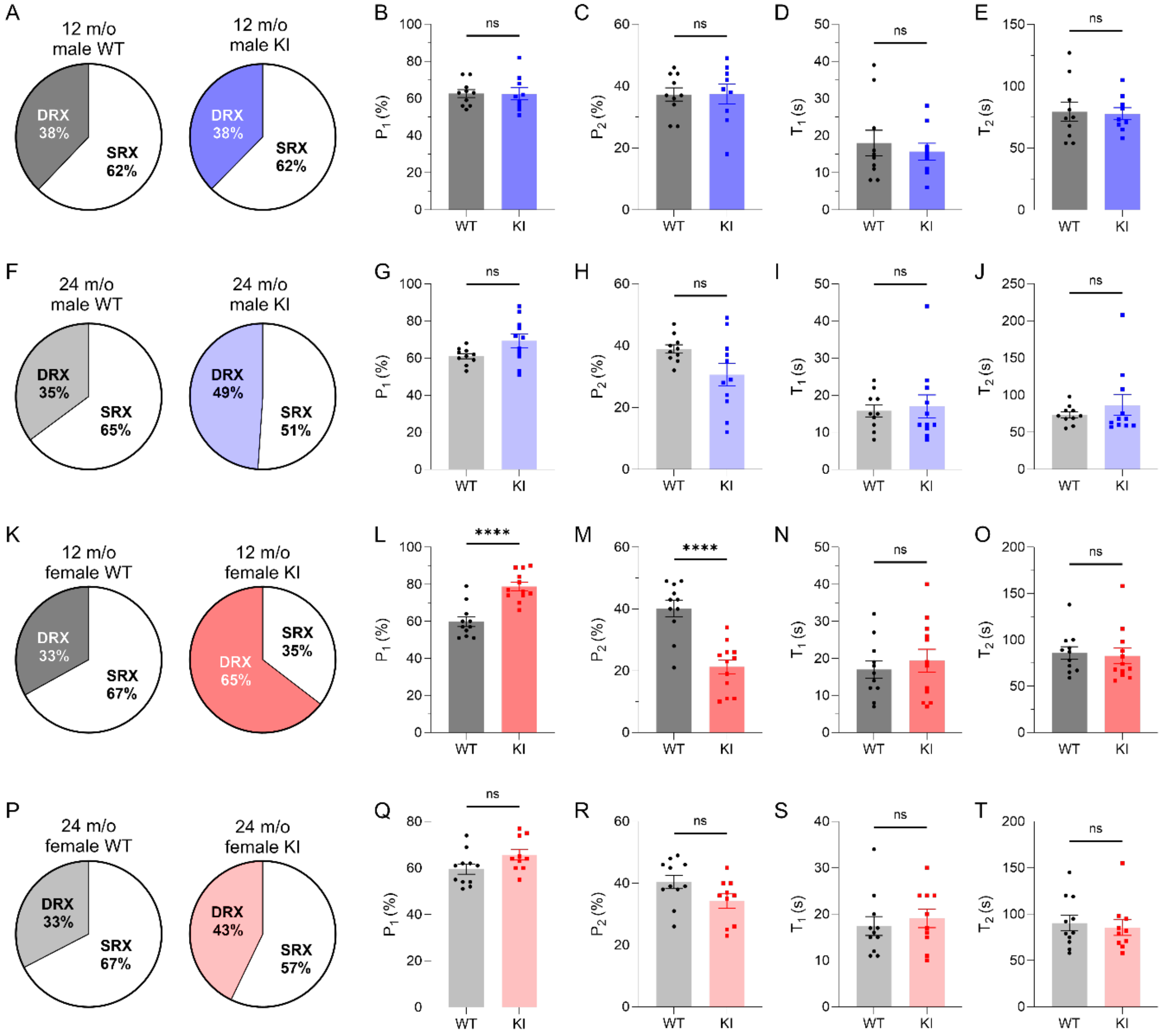

Supplemental Figure 10. Determination of the DRX:SRX myosin ratio in E248K Tibialis Anterior myofibers using single nucleotide turnover Mant-ATP chase assay. The average proportion of myosin heads in the disordered relaxed (DRX) *versus* super relaxed (SRX) state was calculated in both male and female WT and Knock-In (KI) Tibialis Anterior (TA) myofibers at 12- **(A and F)** and 24- **(K and P)** months of age. Individual values of the DRX population (P_1; **B, G, L, and Q)** and lifetime (T_1; **D, I, N, and S)** and the SRX population (P_2; **C, H, M, and R)** and lifetime (T_2; **E, J, O, and T)** were also obtained. WT and KI TA male myofibers exhibit similar DRX:SRX proportions and lifetimes at both 12- **(A-E)** and 24- **(K-O)** months of age. Conversely, KI TA female myofibers contain a significantly higher DRX:SRX ratio compared to WT at 12-months **(F-H)**, which is not sustained though at 24-months **(P-R)**. The lifetimes of the DRX and SRX states remain unchanged at both ages **(I-J and S-T)**; *n*=2 animals per sex/per age/per

genotype and *n'*=9-12 fibers per muscle. Data is presented as mean ± SEM and statistical significance was determined by 2-tailed Student's *t*-test (B-C, E, G-I, and Q-R), with Welch's correction (L and M), and Mann-Whitney test (D, J, N, O, S, and T); ns: not significant, and ****$p < 0.0001$.

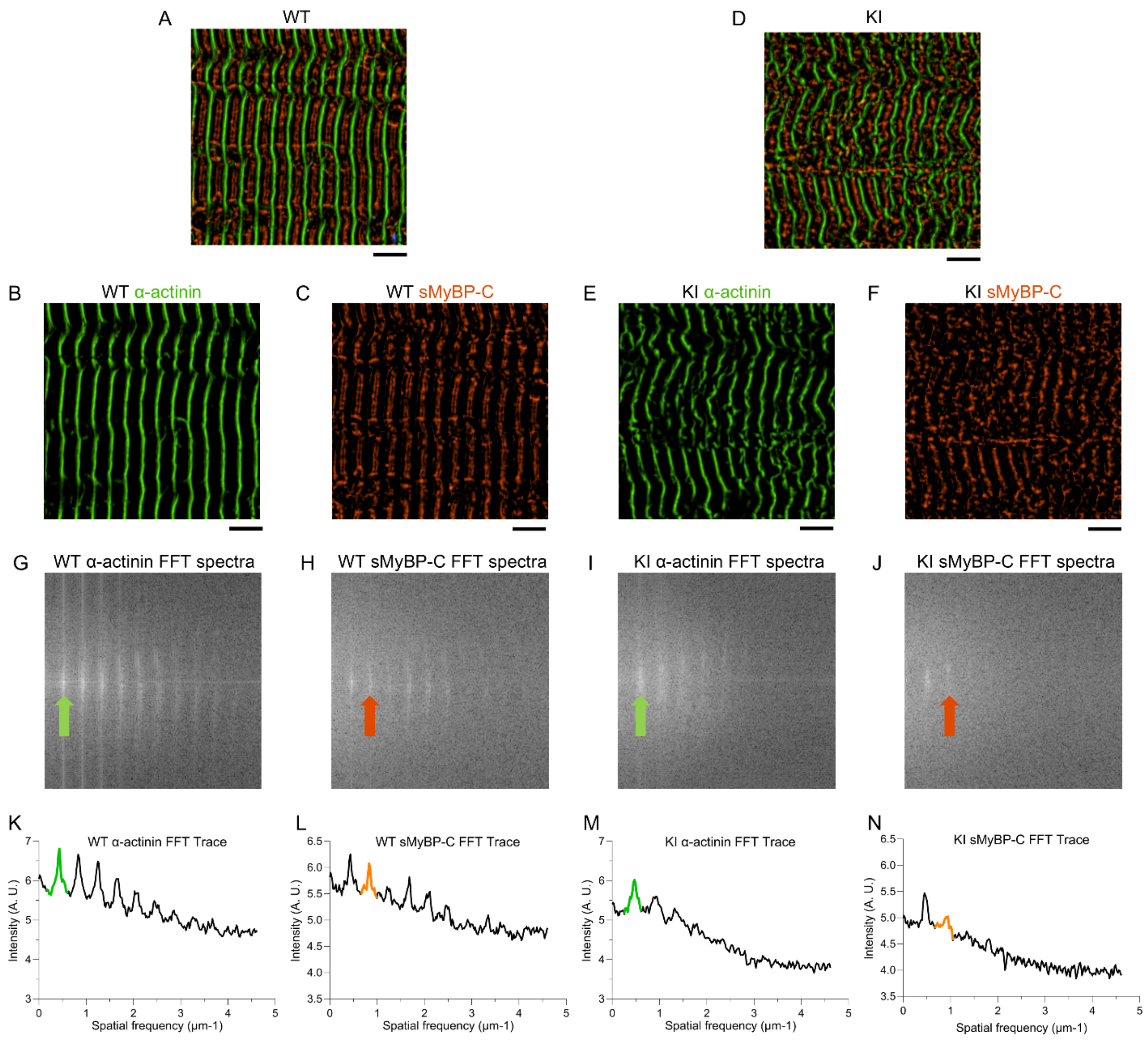

Supplemental Figure 11. Quantification of sarcomeric organization and sMyBP-C localization via FFT analysis. Representative images of tissue sections from 24-month-old male WT **(A-C)** and Knock-In (KI; **D-F)** Tibialis Anterior (TA) muscles co-stained for α-actinin **(B and E)** and slow Myosin Binding Protein-C (sMyBP-C; **C and F)** were used to determine sarcomeric order and sMyBP-C localization scores, respectively; scale bar: 5 μm. To calculate the order score, α-actinin-stained images **(B and E)** underwent Fast Fourier Transform (FFT) analysis followed by generation of the respective power spectra **(G and I)**. The signal intensity along the longitudinal axis was plotted **(K and M)** allowing for visualization of distinct peaks. The first peak, indicated by the green arrow on the power spectra **(G and I)** and marked in green on the FFT traces **(K and M)** corresponds to overall sarcomeric order. Accordingly, the value of the amplitude of the first peak was reported as the order score. Similarly, to determine the sMyBP-C localization score,

sMyBP-C images **(C and F)** underwent FFT analysis **(H and J)**, and the intensity profiles were plotted **(L and N)**. The second peak, indicated by the orange arrow on the power spectra **(H and J)**, and denoted in orange on the FFT traces **(L and N)** corresponds to sMyBP-C localization to the C-zone. Consequently, the value of the amplitude of the second peak was reported as the sMyBP-C localization score.

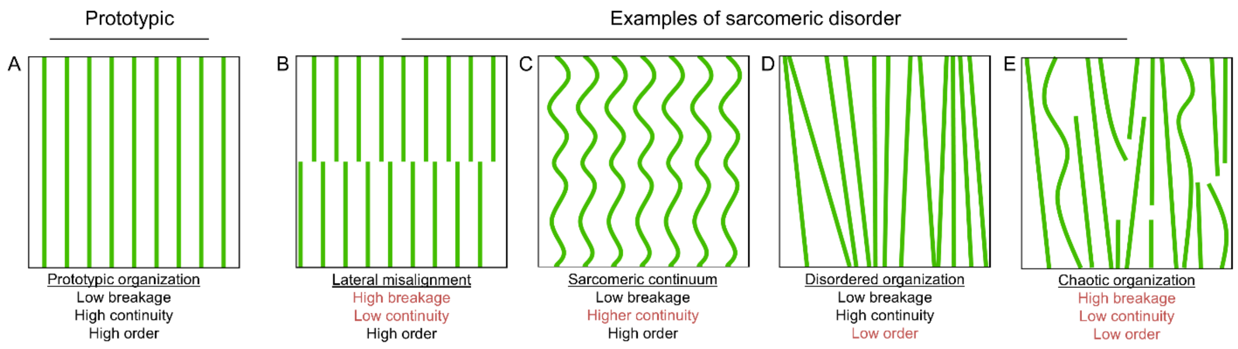

Supplemental Figure 12. Examples of sarcomeric organization metrics. Cartoon representation of sarcomeric organization patterns using α-actinin immunostaining. **(A)** Prototypic sarcomeric organization is characterized by low breakage and high continuity and order scores. **(B-E)** Types of disordered sarcomeric organization and metric changes relatively to the prototypic organization, described as **(B)** lateral misalignment, resulting in increased breakage, decreased continuity, and unchanged order scores; **(C)** sarcomeric continuum, resulting in unchanged breakage and order scores, but increased continuity score; **(D)** disordered organization, resulting in unchanged breakage and continuity scores, but reduced order score; and **(E)** chaotic organization, resulting in higher breakage and lower continuity and order scores. These classifications of sarcomeric (dis)organization were used to deduce the breakage, continuity and order scores described in Figures 7-10 following Fast Fourier Transform (FFT) analysis of α-actinin immunostaining as described in Supplemental Figure 11.

Supplemental Video 1. Behavior of 12-month-old WT male mice. WT male mice show typical exploratory behavior at 12 months, including ambulation, rearing and sniffing without tremor or difficulty.

Supplemental Video 2. Behavior of 12-month-old WT female mice. WT female mice exhibit common exploratory behavior when placed in an isolated cage. Ambulation and rearing are performed in the absence of tremor or difficulty.

Supplemental Video 3. Behavior of 24-month-old WT male mice. 24-month-old WT male mice show slower ambulation when compared to 12-month-old WT mice. However, exploratory behavior is still apparent in the absence of tremor. The observed hair loss is consistent with aging.

Supplemental Video 4. Behavior of 24-month-old WT female mice. 24-month-old female WT mice perform behaviors such as roaming and rearing with no apparent difficulty and in the absence of tremor. Similar to males, hair loss is expected with aging.

Supplemental Video 5. Behavior of 12-month-old E248K Knock In male mice. Observed behavior of 12-month-old male Knock-In (KI) mice when placed in an isolated chamber resembles that of their WT littermates. Mice explore the cage through roaming and rearing events. Evidence of slight gait disturbance and subtle tremor is observed during rearing events. However, gross exploratory function is retained.

Supplemental Video 6. Behavior of 12-month-old E248K Knock In female mice. At 12 months, female Knock-In (KI) mice explore the cage with relative ease. Tremor is apparent with certain actions, such as sniffing. Further, there is evidence of some gait disturbance that reflects mild impairment. However, ambulation and rearing are largely unaffected.

Supplemental Video 7. Behavior of 24-month-old E248K Knock In male mice. At 24-months of age, tremor is apparent in Knock-In (KI) male mice, in multiple areas of the body including the head, abdominal cavity, and limbs, and can be seen upon exertion and occasionally at rest. Rearing becomes more difficult with apparent accompanying limb tremor, and impaired gait, likely due to leg contractures, as well as pronounced kyphosis. Apparent hair loss is a common occurrence with aging.

Supplemental Video 8. Behavior of 24-month-old E248K Knock In female mice. Knock-In (KI) females at 24-months show variable phenotypic severity. Some females are less affected showing evidence of minor gait impairment, likely due to leg contractures, and mild tremor during action. Other females show pronounced kyphosis and intense head and body tremor at rest and with action. Changes in fur coats are expected in response to aging.
