# Supplementary material for: Sex-dependent and muscle-specific progression of the MYBPC1 E248K Myotrem myopathy in response to aging
Source: JCI Insight. 2025 Jun 26;10(15):e182471. doi: 10.1172/jci.insight.182471 (PMC12341543; doi:10.1172/jci.insight.182471)

Full image for Figure 7A; yellow boxes indicate representative ROI

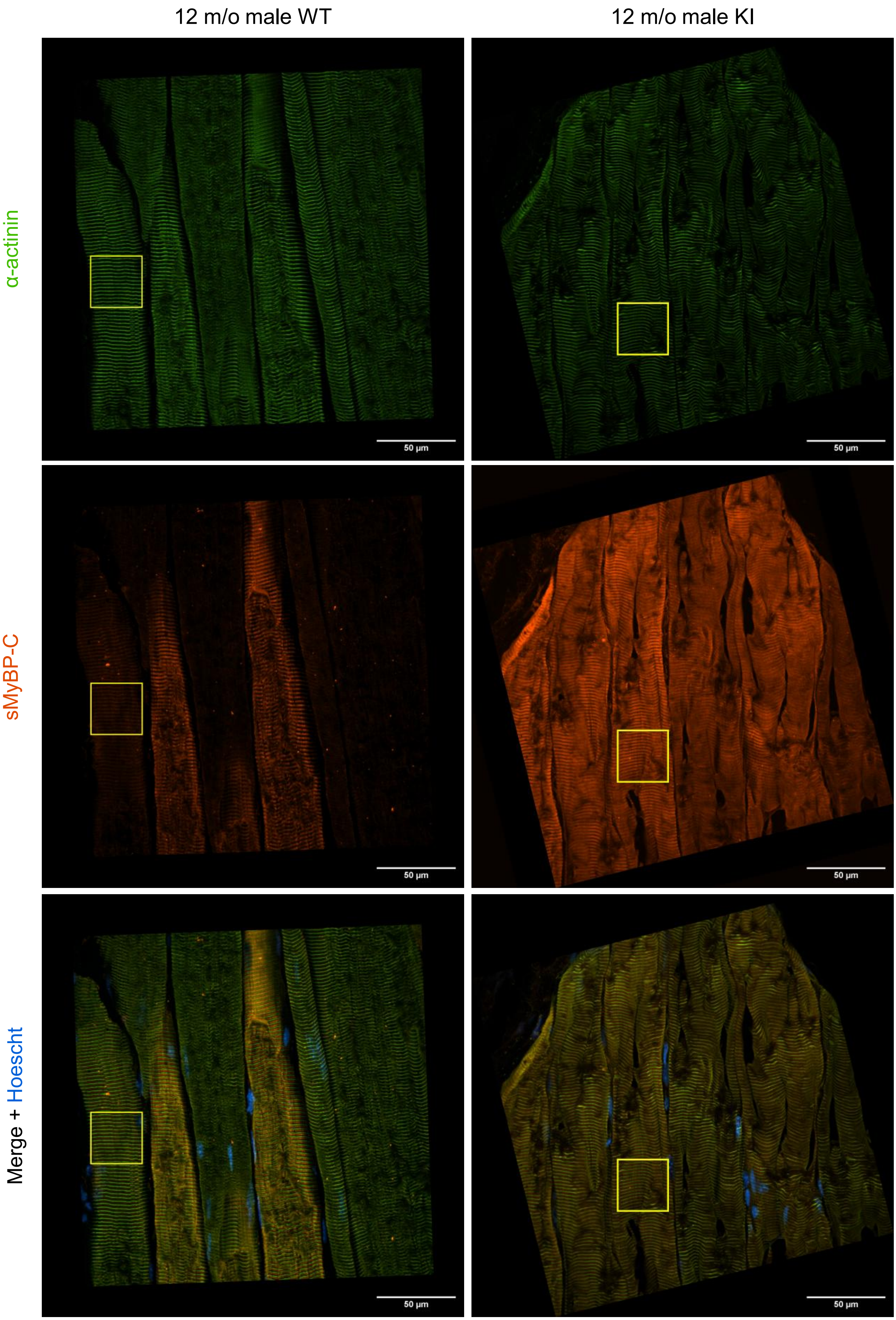

Full image for Figure 8A; yellow boxes indicate representative ROI

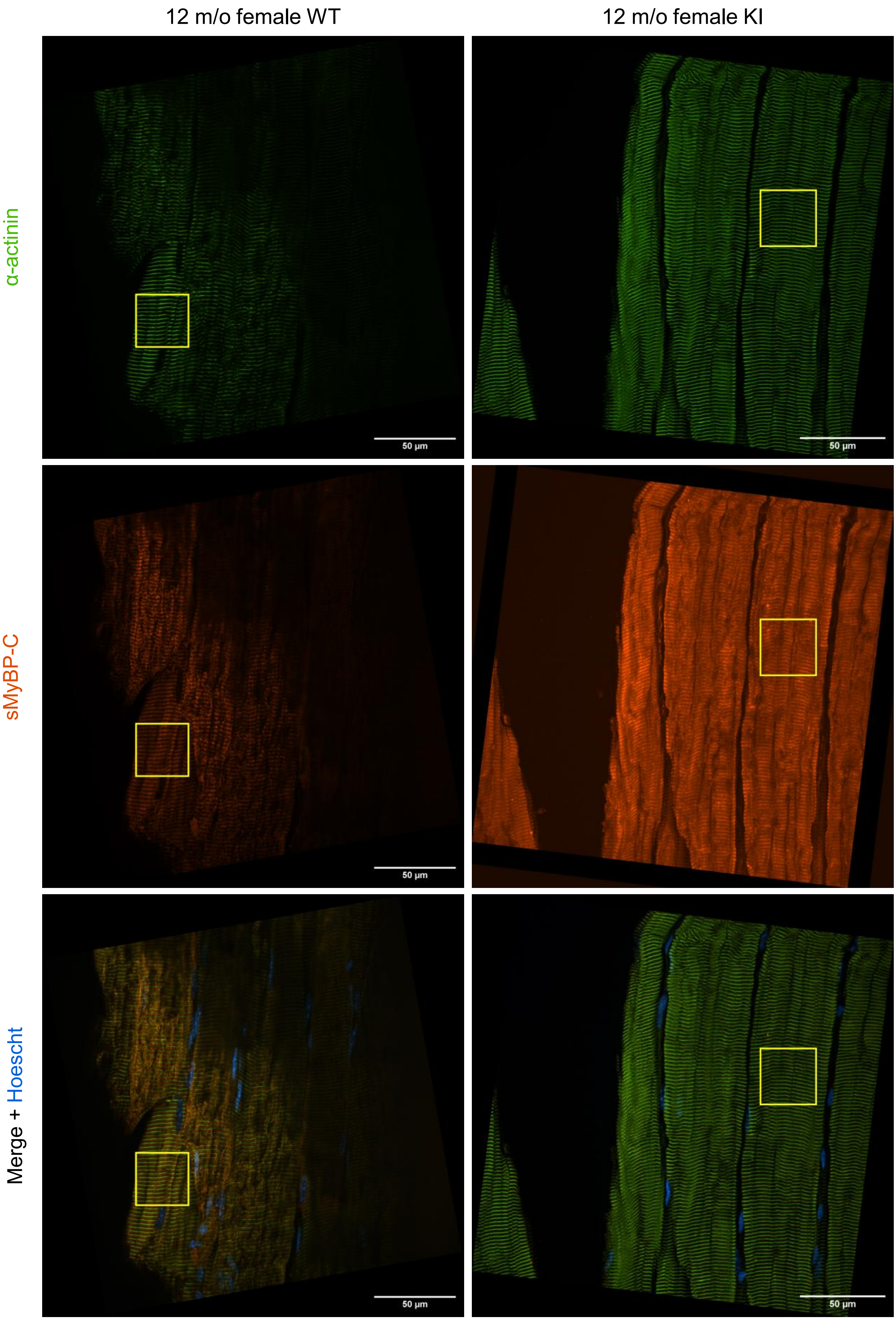

Full image for Figure 9A; yellow boxes indicate representative ROI

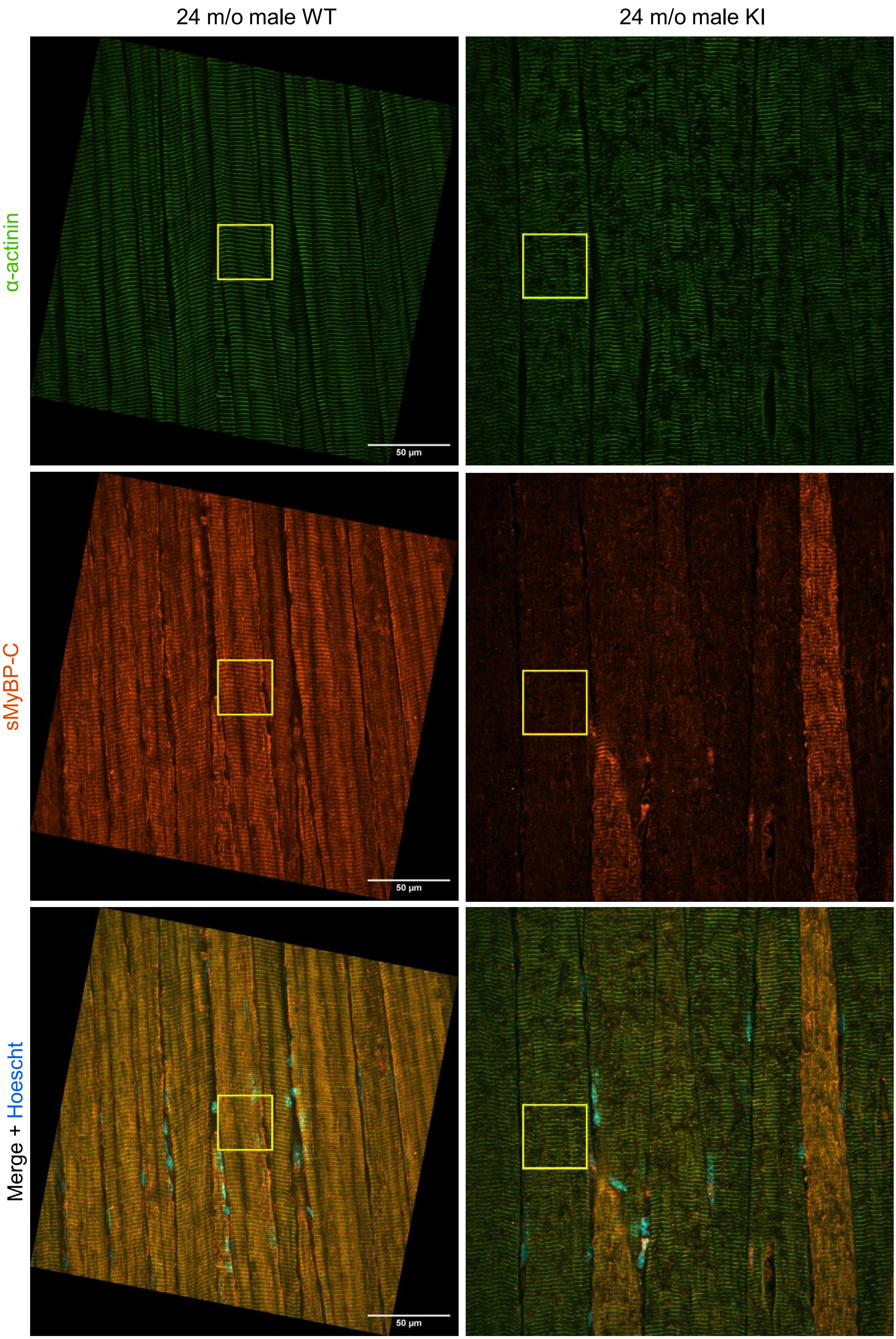

Full image for Figure 10A; yellow boxes indicate representative ROI

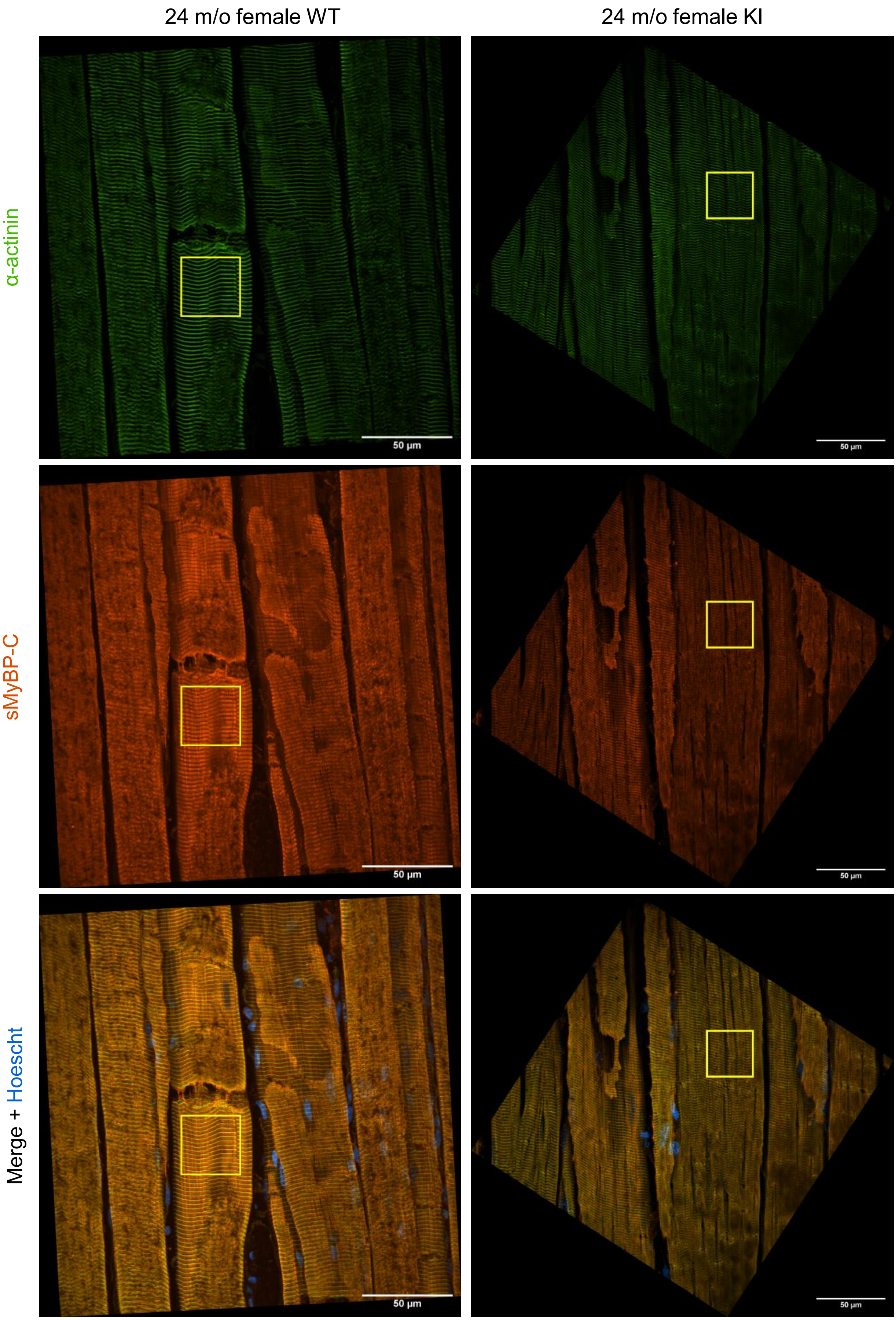

Full unedited Ponceau stain and gel for Supplemental Figure 9A; yellow boxes indicate representative lanes

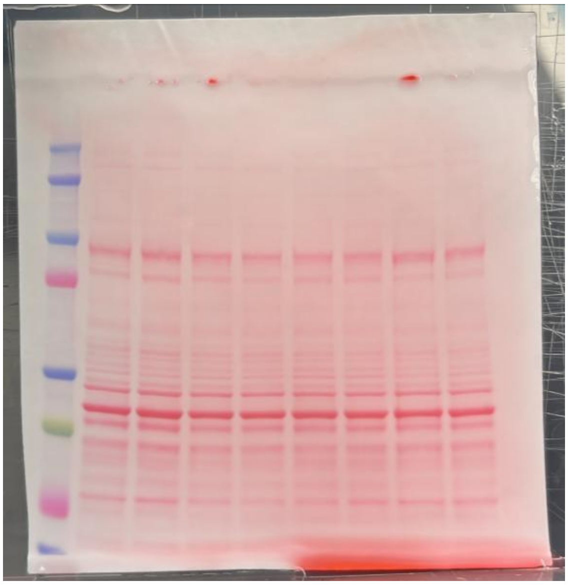

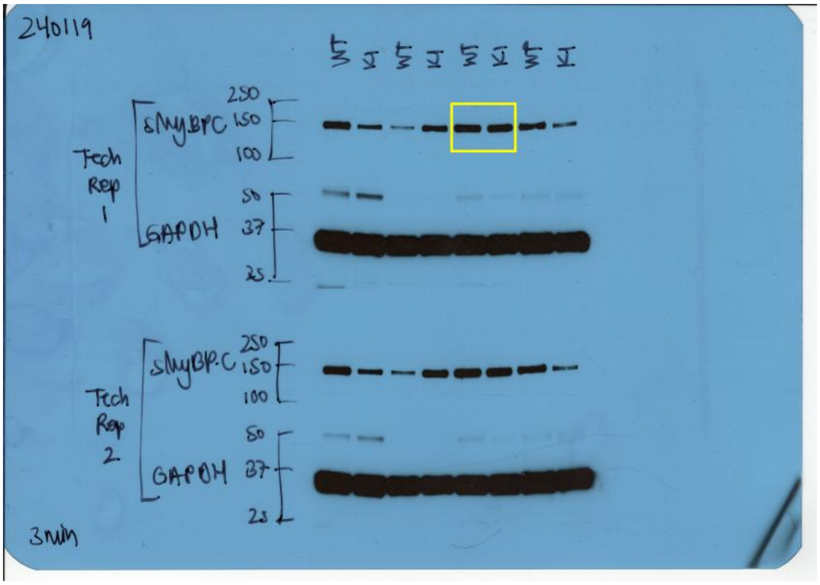


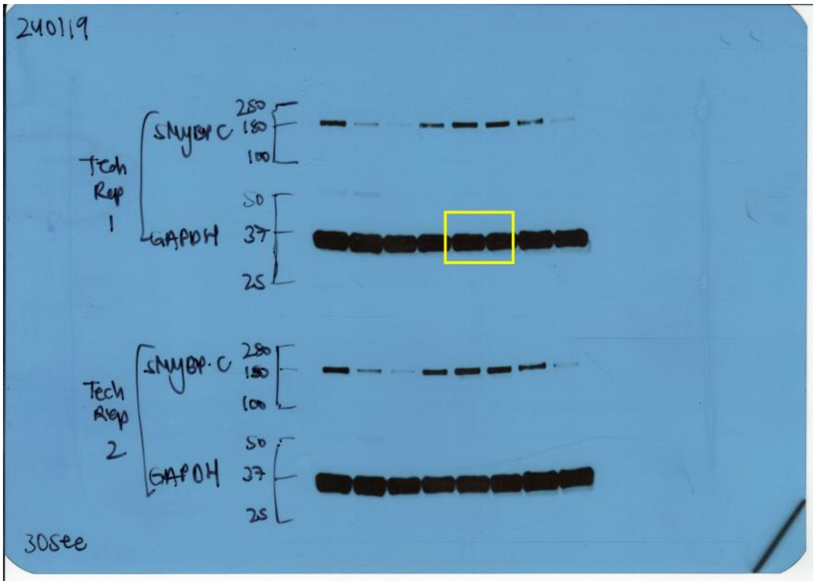

Full unedited Ponceau stain and gel for Supplemental Figure 9B; yellow boxes indicate representative lanes

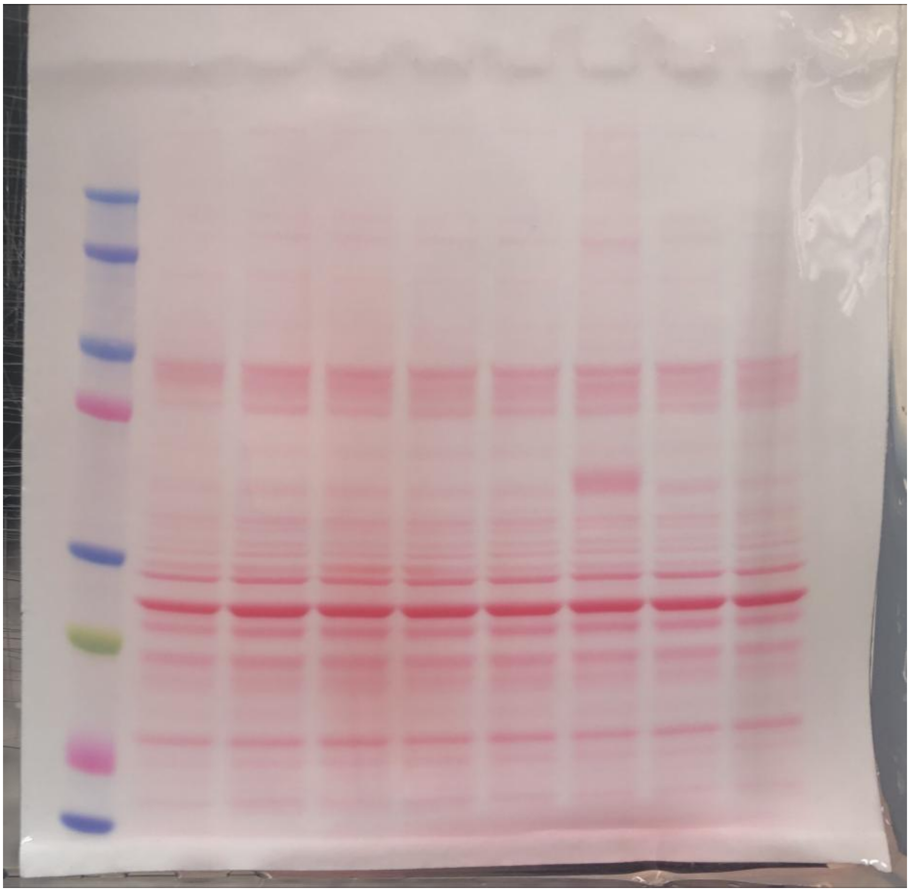

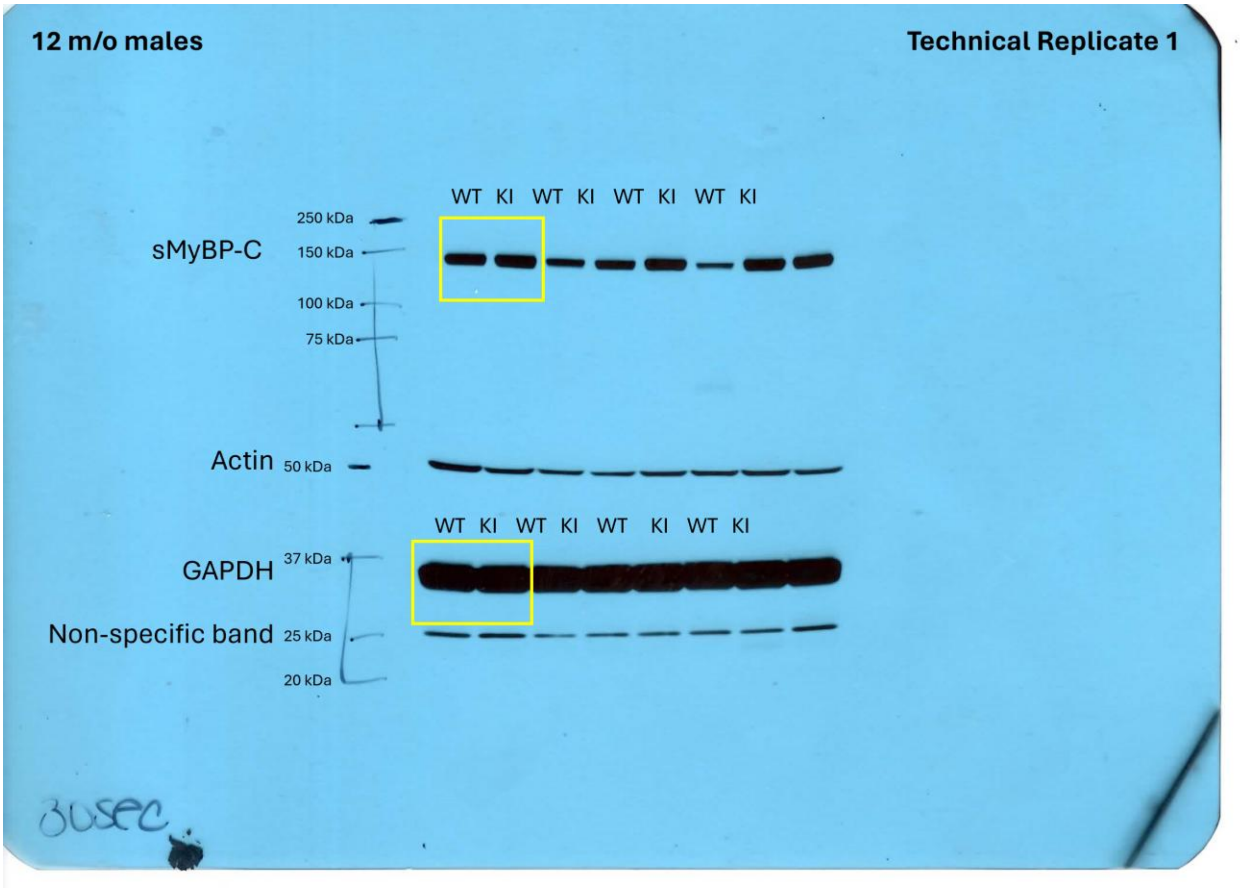

Full unedited Ponceau stain and gel for Supplemental Figure 9C; yellow boxes indicate representative lanes

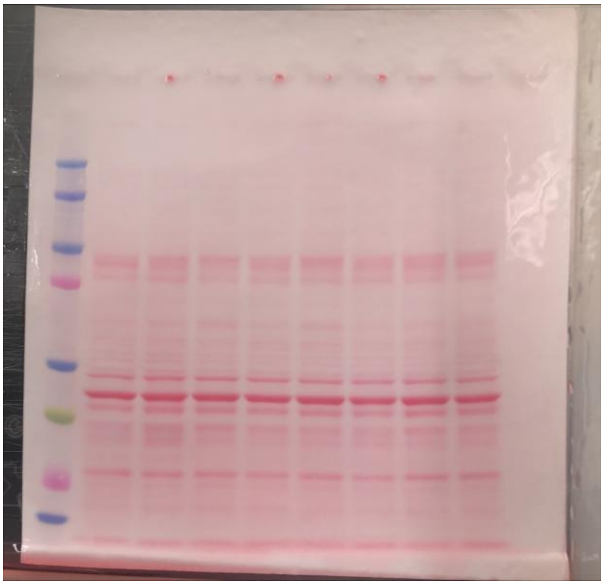

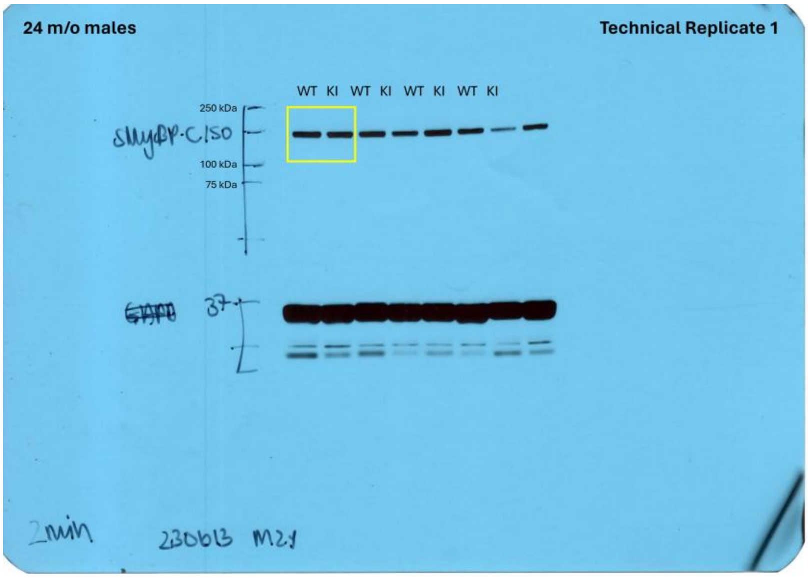


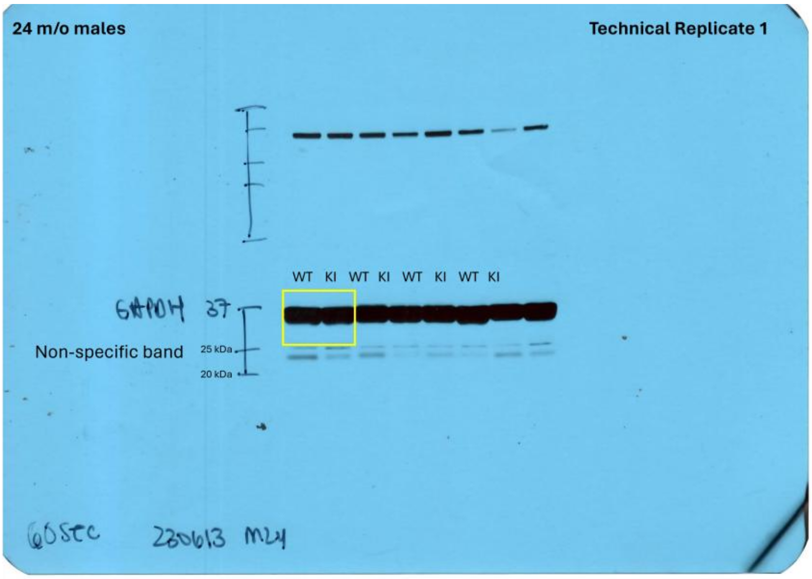

Full unedited Ponceau stain and gel for Supplemental Figure 9D; yellow boxes indicate representative lanes

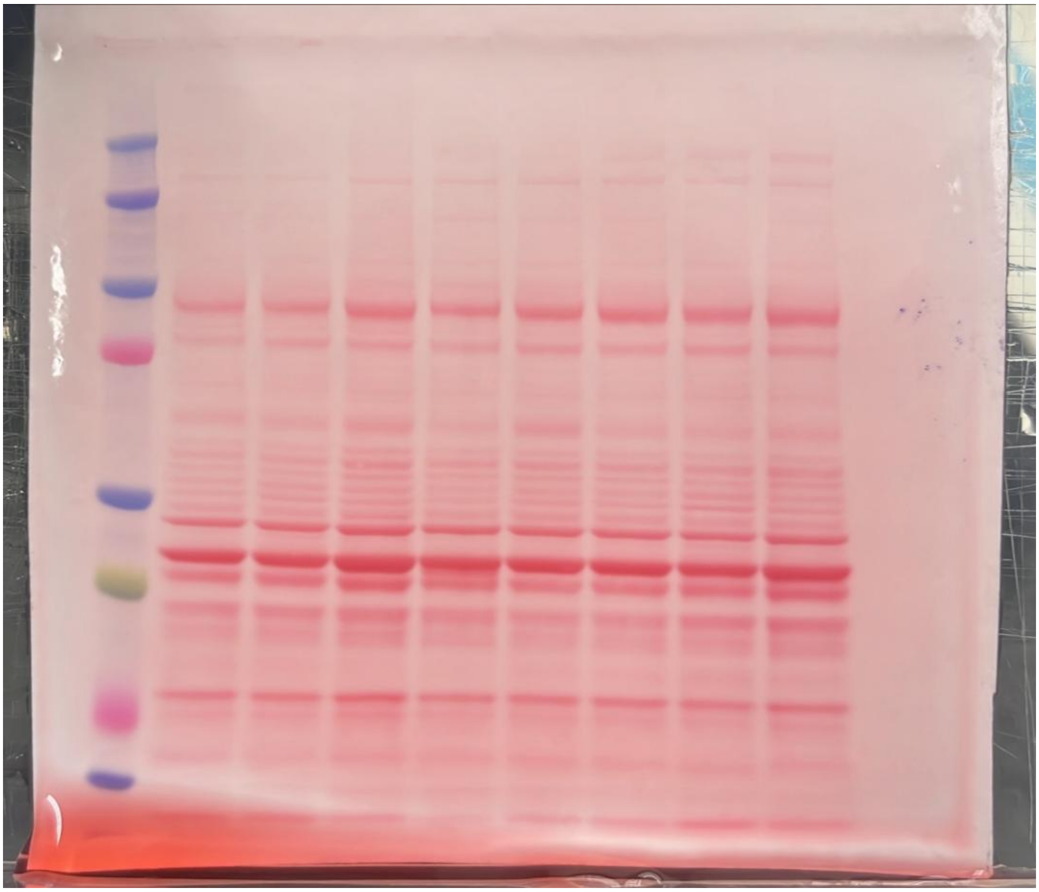

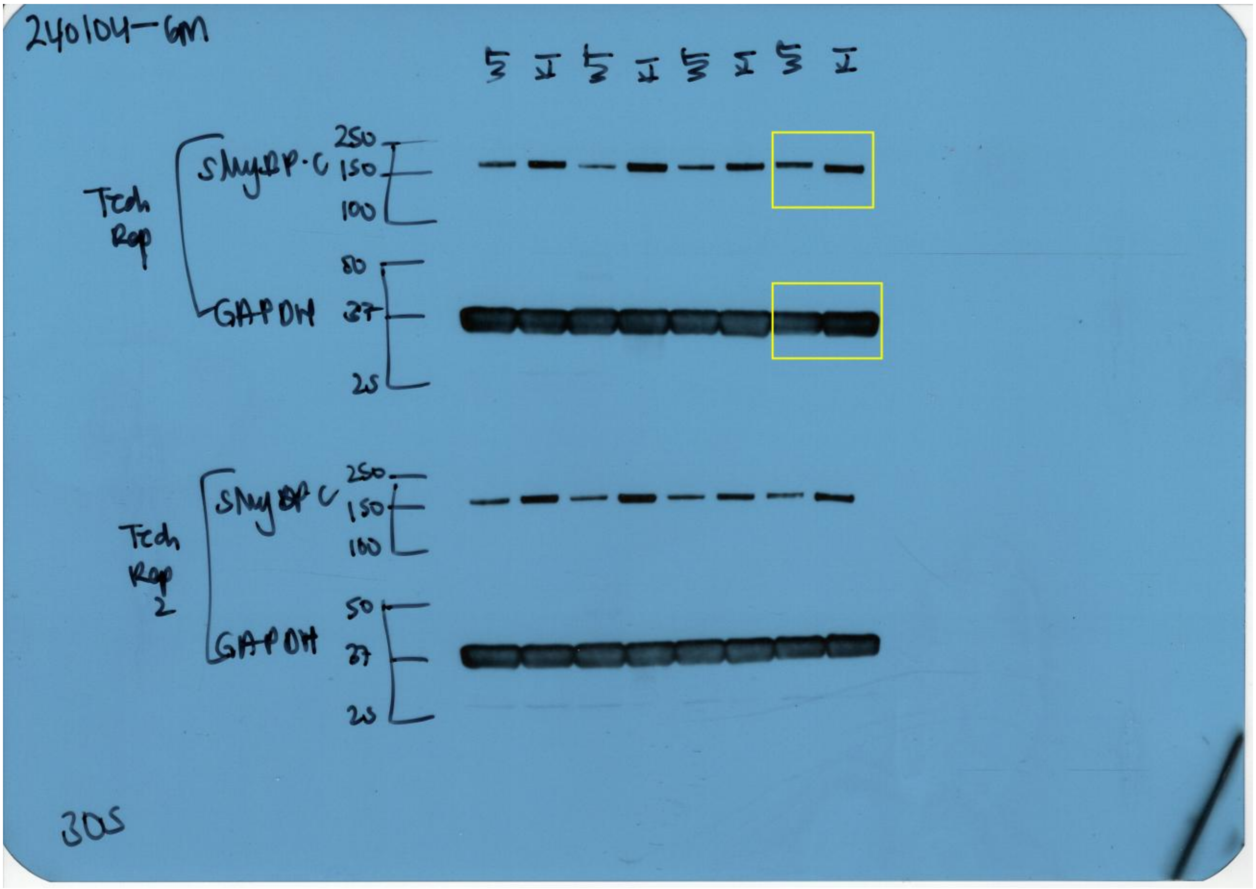

Full unedited Ponceau stain and gel for Supplemental Figure 9E; yellow boxes indicate representative lanes

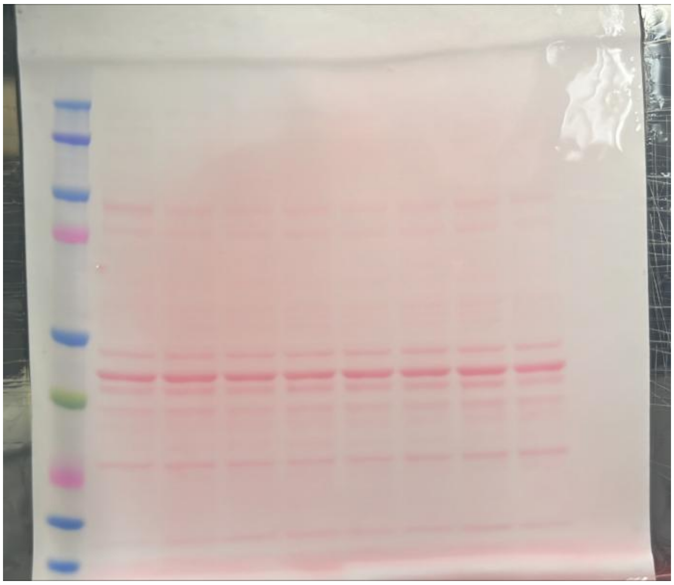

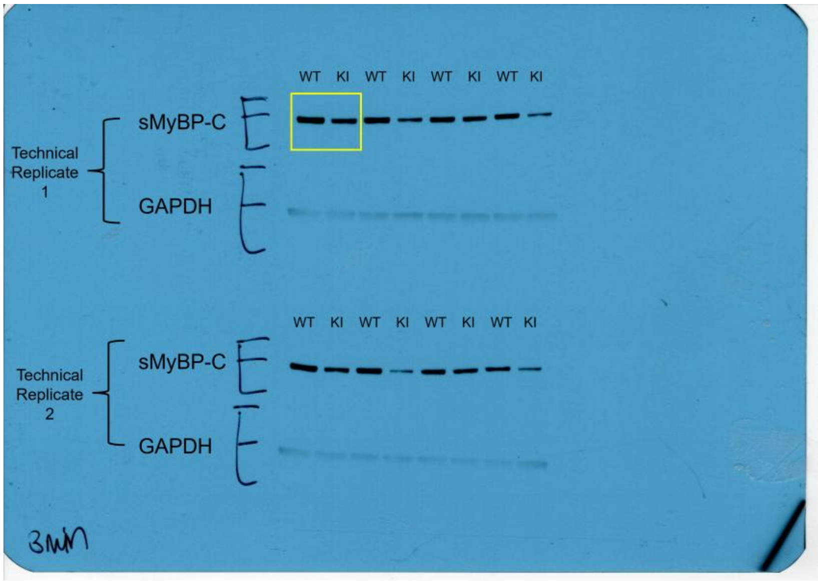


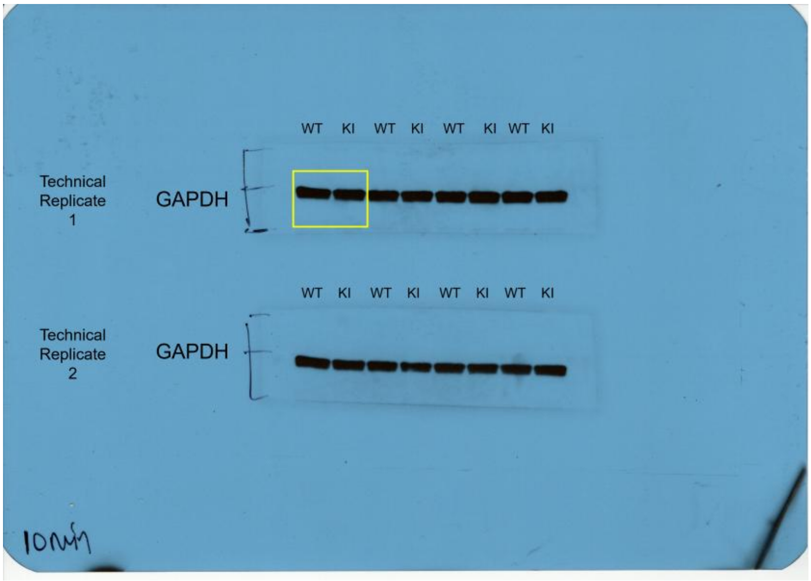

Full unedited Ponceau stain and gel for Supplemental Figure 9F; yellow boxes indicate representative lanes

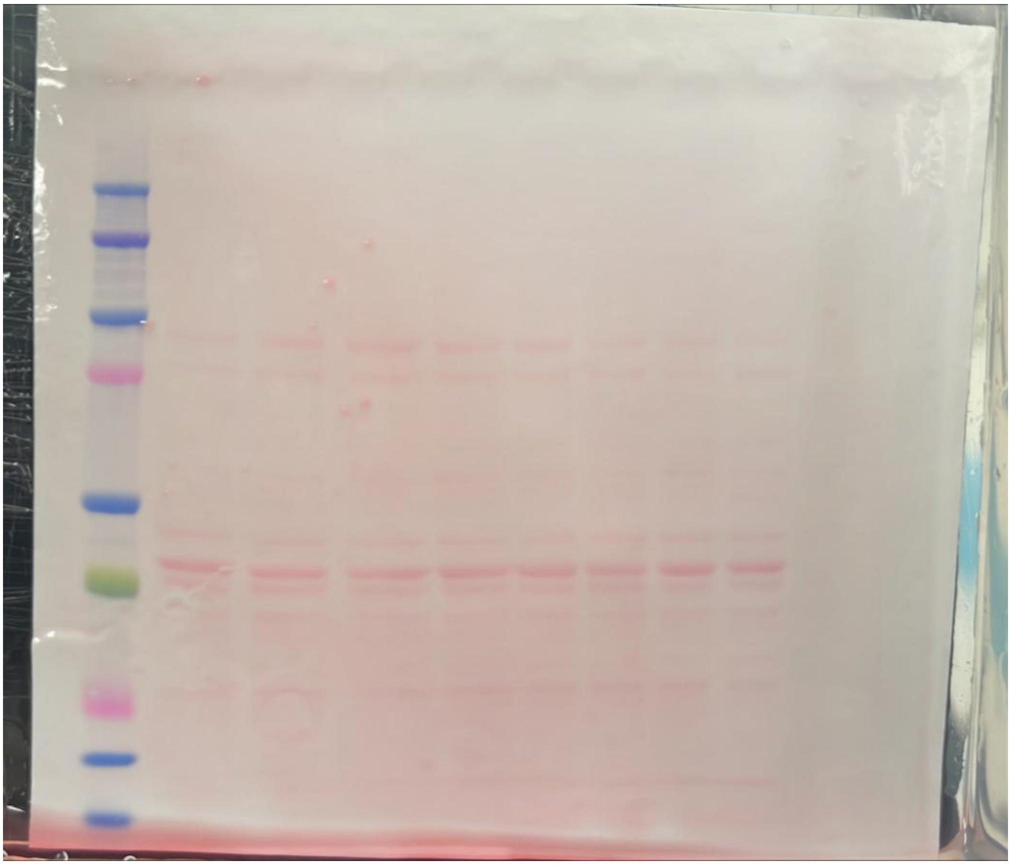

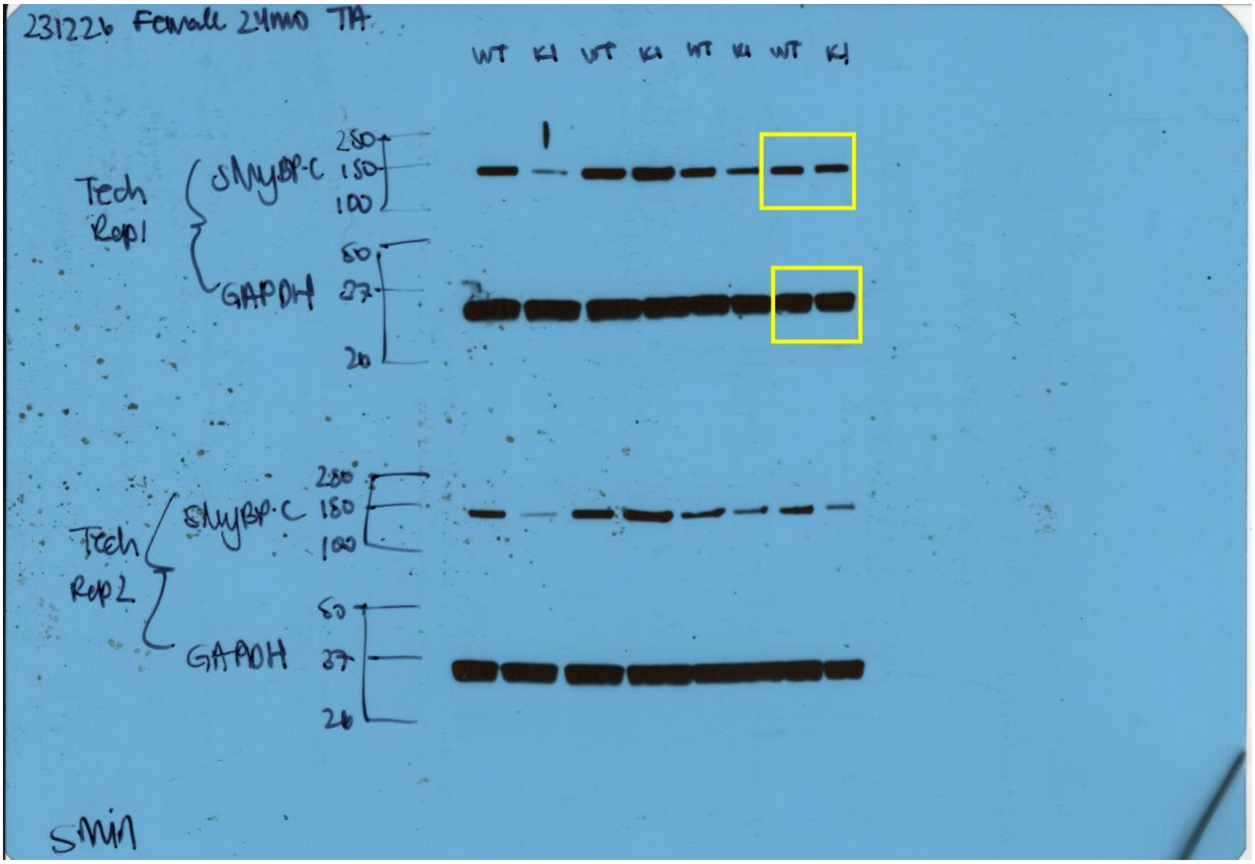

Full images for Supplemental Figure 11; yellow boxes indicate representative ROI

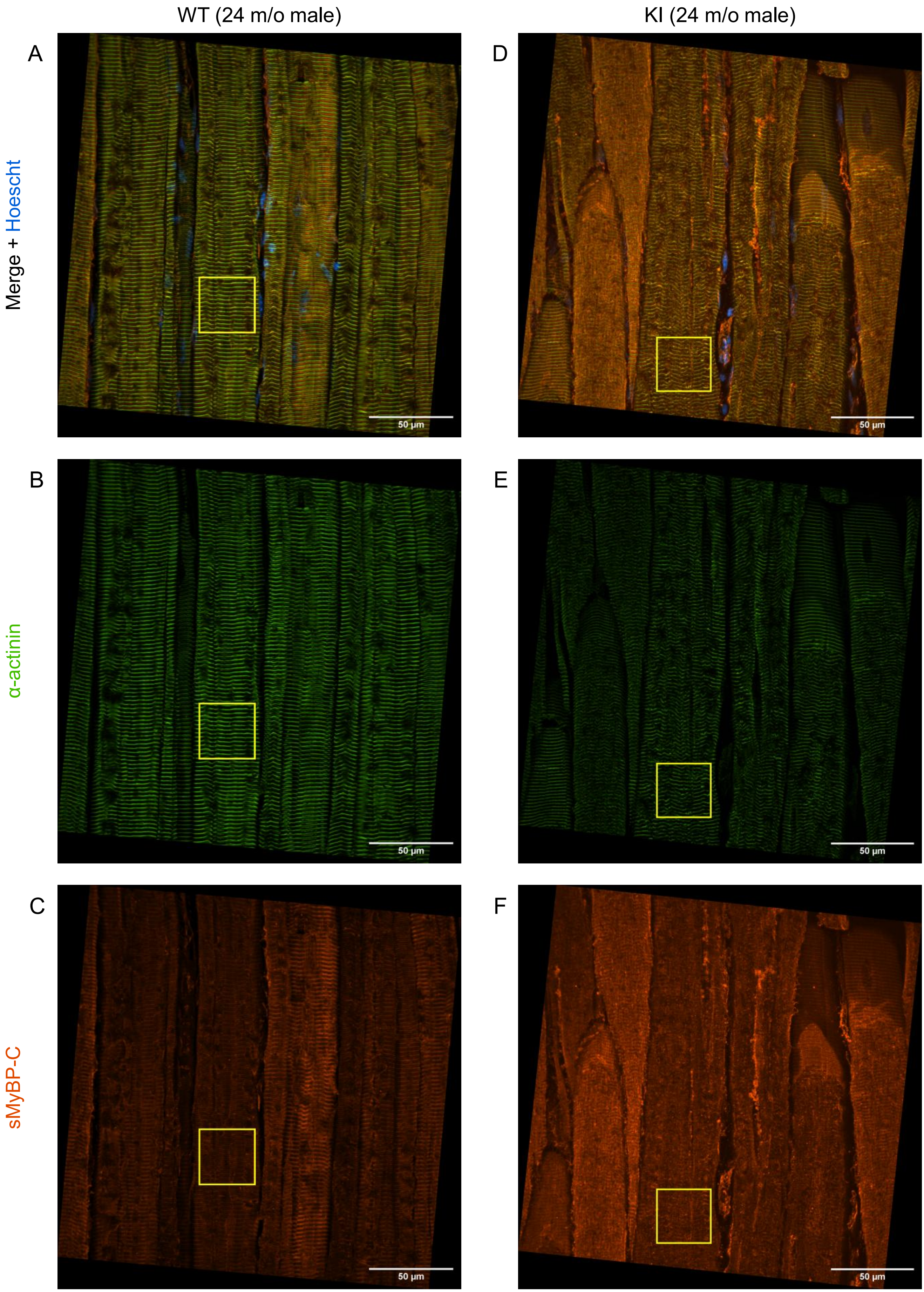

Supplement: Unedited blot and gel images [file jciinsight-10-182471-s024.pdf]
